# Passenger Gene Coamplifications Create Collateral Therapeutic Vulnerabilities in Cancer

**DOI:** 10.1158/2159-8290.CD-23-1189

**Published:** 2024-01-13

**Authors:** Yi Bei, Luca Bramé, Marieluise Kirchner, Raphaela Fritsche-Guenther, Severine Kunz, Animesh Bhattacharya, Mara-Camelia Rusu, Dennis Gürgen, Frank P.B. Dubios, Julia K.C. Köppke, Jutta Proba, Nadine Wittstruck, Olga Alexandra Sidorova, Rocío Chamorro González, Heathcliff Dorado Garcia, Lotte Brückner, Robin Xu, Mădălina Giurgiu, Elias Rodriguez-Fos, Qinghao Yu, Bastiaan Spanjaard, Richard P. Koche, Clemens A. Schmitt, Johannes H. Schulte, Angelika Eggert, Kerstin Haase, Jennifer Kirwan, Anja I.H. Hagemann, Philipp Mertins, Jan R. Dörr, Anton G. Henssen

**Affiliations:** 1 Department of Pediatric Oncology/Hematology, Charité-Universitätsmedizin Berlin, Berlin, Germany.; 2 German Cancer Consortium (DKTK), Partner Site Berlin, and German Cancer Research Center (DKFZ), Heidelberg, Germany.; 3 Core Unit Proteomics, Berlin Institute of Health at Charité-Universitätsmedizin Berlin and Max Delbrück Center for Molecular Medicine, Berlin, Germany.; 4 Core Unit Metabolomics, Berlin Institute of Health at Charité-Universitätsmedizin Berlin, Berlin, Germany.; 5 Max-Delbrück-Center for Molecular Medicine in the Helmholtz Association (MDC), Technology Platform Electron Microscopy, Berlin, Germany.; 6 Department of Hematology, Oncology and Tumor Immunology, Charité-Universitätsmedizin Berlin, Berlin, Germany.; 7 Experimental Pharmacology and Oncology (EPO), Berlin, Germany.; 8 Institute of pathology, Charité-Universitätsmedizin Berlin, Corporate Member of Freie Universität Berlin, Humboldt-Universität zu Berlin, Berlin, Germany.; 9 Center for Epigenetics Research, Memorial Sloan Kettering Cancer Center, New York, New York.; 10 Berlin Institute of Health, Berlin, Germany.; 11 Experimental and Clinical Research Center (ECRC) of the MDC and Charité Berlin, Berlin, Germany.

## Abstract

DNA amplifications in cancer do not only harbor oncogenes. We sought to determine whether passenger coamplifications could create collateral therapeutic vulnerabilities. Through an analysis of >3,000 cancer genomes followed by the interrogation of CRISPR-Cas9 loss-of-function screens across >700 cancer cell lines, we determined that passenger coamplifications are accompanied by distinct dependency profiles. In a proof-of-principle study, we demonstrate that the coamplification of the *bona fide* passenger gene DEAD-Box Helicase 1 (*DDX1*) creates an increased dependency on the mTOR pathway. Interaction proteomics identified tricarboxylic acid (TCA) cycle components as previously unrecognized DDX1 interaction partners. Live-cell metabolomics highlighted that this interaction could impair TCA activity, which in turn resulted in enhanced mTORC1 activity. Consequently, genetic and pharmacologic disruption of mTORC1 resulted in pronounced cell death *in vitro* and *in vivo*. Thus, structurally linked coamplification of a passenger gene and an oncogene can result in collateral vulnerabilities.

**Significance:**

We demonstrate that coamplification of passenger genes, which were largely neglected in cancer biology in the past, can create distinct cancer dependencies. Because passenger coamplifications are frequent in cancer, this principle has the potential to expand target discovery in oncology.

*This article is featured in Selected Articles from This Issue, p. 384*

## Teaser

Passenger gene co-amplification is a largely overlooked but common event in many cancers that creates tumor-specific, therapeutically actionable dependencies with the potential to expand target discovery in oncology.

## INTRODUCTION

Somatic DNA amplification is a common phenomenon in cancer and represents one of the most important causes of excessive oncogene expression (1). To date, many oncogene amplifications remain therapeutically unactionable. Recent cancer genome sequencing studies have revealed fundamental insights into amplicon structures (2–4). Their results demonstrated that DNA amplifications exist in at least two forms: (i) self-repeating arrays on a chromosome (homogeneously staining regions, HSR) and (ii) circular extrachromosomal DNA (ecDNA; ref. 4). The genomic boundaries of such amplicons are not randomly distributed around oncogenes but are largely defined by the location of nearby core-regulatory enhancer elements (2, 3). Enhancers that are included on amplicons are required for sustained oncogene expression (2, 3, 5, 6), suggesting that genomic regions coamplified with oncogenes are under strong positive selection. These emerging structural properties of DNA amplicons may explain the recurrently observed coamplification of passenger genes in the vicinity of the amplified oncogene. The fact that such passenger genes are retained on amplicons in cancer cells implies that their amplification does not compromise cancer cell survival. Whether and how passenger gene coamplifications alter cancer cell physiology, however, has not been investigated conclusively to date.

Inspired by the concept of “collateral lethality,” which enabled the identification of cancer-specific therapeutic vulnerabilities resulting from codeletions of genes neighboring tumor suppressor genes (7), we here investigated whether passenger gene coamplification could present novel therapeutic strategies for tumors harboring oncogene amplifications, for which drug development has remained largely elusive to date.

## RESULTS

### Passenger Gene Coamplifications Are Associated with Distinct Pathway Dependencies across Cancers

**Figure 1.**
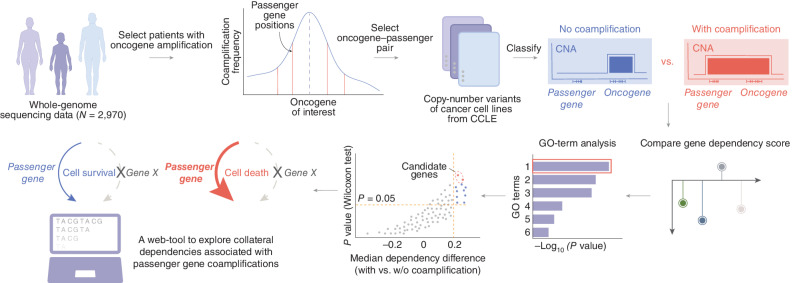
Overview of the systematic approach to identify collateral lethal dependencies associated with passenger gene coamplifications in cancers.

**Figure 2.**
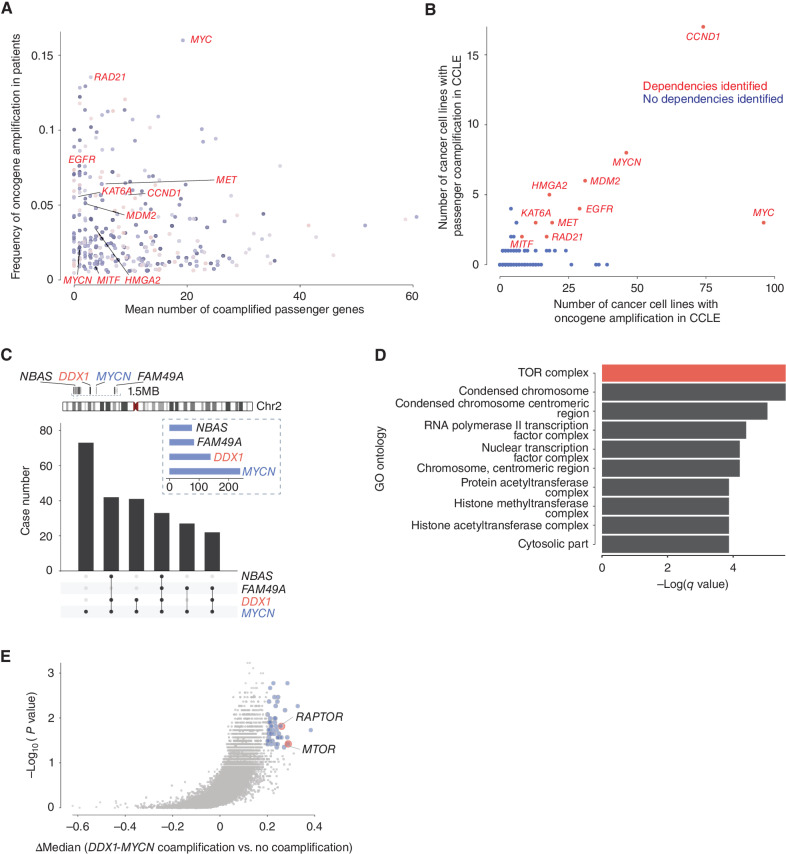
Passenger genes are frequently coamplified with oncogenes in cancers and can be associated with distinct dependencies. **A,** The mean number of passenger genes coamplified with oncogenes compared with the frequency of oncogene amplifications in cancers (dot size presents incidence passenger gene coamplification). **B,** Number of cancer cell lines in CCLE with an oncogene amplification compared with the number of cell lines with passenger coamplification (oncogenes for which sufficient dependency information is available in the DepMap are marked in red). **C,** Chromosome 2 schematic highlighting the area of *MYCN* amplification and passenger genes recurrently included on the amplicon (top). Upset plot (bottom) for the coamplification frequency of three passenger genes, *NBAS*, *DDX1*, and *FAM49A*, identified on the *MYCN* amplicon in a cohort of 556 neuroblastomas. **D,** Top 10 GO ontology terms enriched for collateral lethal targets associated with *DDX1*-*MYCN* coamplifications (TOR complex labeled in red). **E,** Difference in gene dependency scores between cancer cell lines with *DDX1*-*MYCN* coamplification vs. cell lines with *MYCN* amplifications compared with the log-transformed *P* values (Wilcoxon; candidate collateral lethal dependencies in *DDX1*-*MYCN* coamplified cancer cells, blue; mTORC1 complex, red).

The recent discovery that oncogene amplifications encompass large neighboring genomic regions with regulatory elements suggests that coamplification of nearby passenger genes may be more common in cancer than previously anticipated. To assess the frequency of passenger gene coamplification, we analyzed whole-genome sequences from well-characterized cohorts of childhood and adult tumors (8–10), respectively. Passenger gene amplifications were common in all analyzed tumor entities (Figs. 1 and 2A; Supplementary Fig. S1A and S1B) but very rarely occurred on amplicons not harboring oncogenes, raising the possibility that their coamplification may generate collateral therapeutic vulnerabilities.

To identify dependencies specifically associated with passenger gene coamplifications, we analyzed copy-number profiles of human cancer cell lines from the Broad-Novartis Cancer Cell line Encyclopedia (CCLE; refs. 11, 12) and selected all cancer cell lines with oncogene amplifications. Next, we compared the genetic dependencies of cell lines with passenger gene coamplifications with those only harboring oncogene amplifications by analyzing genome-scale pooled CRISPR/Cas9 loss of viability screens in >700 genomically characterized human cancer cells from 26 tumor lineages as part of the Cancer Dependency Map (ref. 13, bioRxiv 2019.06.31.720243). We calculated the dependency scores as well as their median difference for each gene between cell lines with oncogene amplifications versus cell lines with additional passenger coamplifications. Significant differences in dependencies between these groups were identified for 10 out of 318 oncogenes (Fig. 2B), e.g., *MYC* and *EGFR* (Supplementary Fig. S1C–S1H). All passenger coamplification-associated dependencies were made available in an openly accessible web portal (https://github.com/yeebae1118/PassengerDepMap).

### *DDX1* Is Frequently Coamplified with *MYCN* in Cancers and Is Accompanied by a Collateral mTORC1 Dependency

To uncover how far collateral vulnerabilities can be directly induced by coamplified passenger genes, we performed a proof-of-principle analysis in models with *MYCN* amplifications, which are frequent in many tumor entities and are often associated with high-risk disease and poor therapeutic outcome (14). As a basic helix–loop–helix oncogenic transcription factor, *MYCN* remains inapproachable for direct therapeutic interventions, making it an ideal and clinically highly relevant candidate to test our hypothesis. Analysis of 556 published neuroblastoma genome-wide copy-number profiles (15, 16) identified 238 neuroblastomas with *MYCN* amplifications (Fig. 2C). In line with our previous work (3), the *MYCN* amplicon on average encompassed a large 1–3 Mb region with several coamplified passenger genes, including *DDX1*, *NBAS*, and *FAM49A*. As suggested by other reports (17, 18), *DDX1*, a gene encoding for the Asp-Glu-Ala-Asp (DEAD) functioning ATPase DDX1 (17, 19), was the most recurrently coamplified passenger gene with *MYCN* in neuroblastomas (57.98%, 138 out of 238; Fig. 2C; Supplementary Fig. S1I). No *DDX1* amplifications without *MYCN* were detectable in 556 cancer genomes. *DDX1-MYCN* coamplification also occurred in several other cancer entities (Supplementary Fig. S1J). Combined analysis of copy-number and mRNA expression of *DDX1* and *MYCN* confirmed a significant positive correlation in expression and aberrantly high *DDX1* expression levels in the context of coamplification (Supplementary Fig. S1K–S1L and Supplementary Fig. S2A–S2G). Consistently, neuroblastoma cell lines harboring *DDX1*-*MYCN* coamplification had elevated DDX1 protein and mRNA levels compared with those lacking *DDX1* coamplifications or cells without *MYCN* amplification (Supplementary Fig. S2H). Thus, *DDX1*-*MYCN* coamplification is present in a considerable fraction of cancers and is associated with aberrantly high DDX1 expression, which could affect cancer cell physiology.

**Figure 3.**
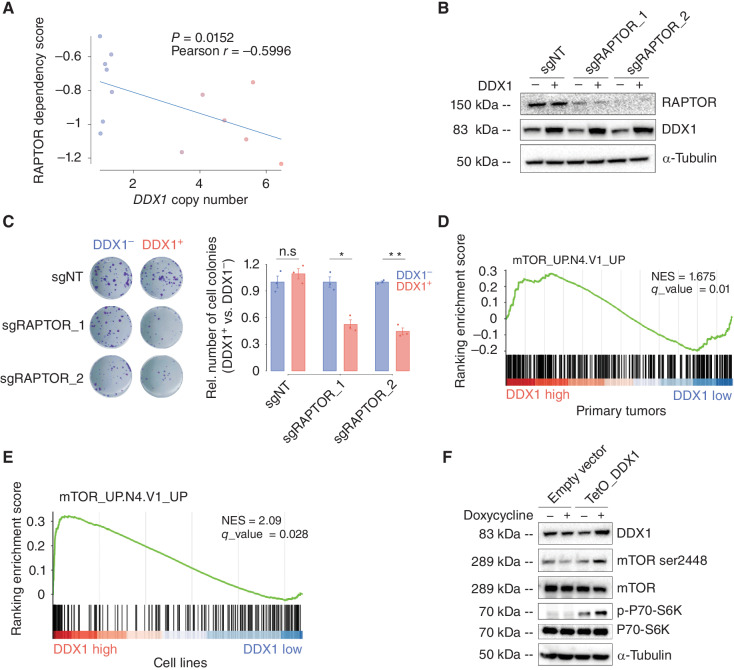
A proof-of-principle study identifies a selective mTOR pathway dependency in cells with *DDX1-MYCN* coamplification. **A,** Correlation between *DDX1* copy-number and dependency scores (CERES) for *RAPTOR* in neuroblastoma cell lines (Pearson correlation analysis, *R* = −0.5996, *P* = 0.0152, *N* = 13). **B,** Western immunoblot of RAPTOR and DDX1 in the KELLY cells transduced with the doxycycline-inducible DDX1-mCherry vectors and with two pairs of sgRNAs targeting *RAPTOR (*sgRAPTOR) or a nontargeting sgRNA (sgNT) as well as Cas9 in the presence and absence of doxycycline (1 μg/mL). Tubulin serves as a loading control. **C,** Representative images of cell colonies formed by KELLY cells transduced with the doxycycline-inducible DDX1-mCherry vectors and with two pairs of sgRNA targeting *RAPTOR* (sgRAPTOR) or nontarget sgRNA (sgNT) as well as Cas9 in the presence and absence of doxycycline (1 μg/mL) and stained with crystal violet (left). Quantification of colony numbers (right, mean ± SE. *N* = 3 biological replicates; Welch *t* test, *P* = 0.564, 0.000117, and 0.00131 for sgNT, sgRAPTOR_1, and sgRAPTOR_2, respectively). **D,** Gene set enrichment analysis (GSEA) based on a set of genes regulated by mTORC1 measured in genes differentially expressed in tumors with high versus low DDX1 expression. **E,** GSEA based on a set of genes regulated by mTORC1 measured in genes differentially expressed in KELLY cells harboring a *MYCN* amplification with versus without ectopic DDX1 expression. **F,** Western blot of the relative protein expression of mTOR ser2448 phosphorylation and P70-S6K Thr389 phosphorylation in KELLY cell after inducible expression of DDX1 (1,000 ng/mL doxycycline treatment for 48 hours).

Our collateral dependency analysis (Fig. 1) revealed that *DDX1*-*MYCN* coamplification was significantly associated with high genetic dependency to several pathways and particularly to mTORC1 complex members mTOR and its scaffold protein RAPTOR, which plays an important role in mTORC1 activation (ref. 20; Fig. 2D and E; Supplementary Fig. S3A and S3B; Supplementary Table S1). In line with an increased RAPTOR dependency of cells with a *DDX1*-*MYCN* coamplification, dependency scores for RAPTOR significantly negatively correlated with the *DDX1* copy-number in *MYCN*-amplified neuroblastoma cell lines (Fig. 3A, Pearson coefficient = −0.5996, *P* = 0.0152; Supplementary Fig. S3C and S3D). Moreover, ectopic expression of DDX1 in *MYCN*-amplified cell lines was sufficient to increase RAPTOR dependency, as evidenced by reduced clonogenicity of cells after CRISPR-Cas9-mediated knockout of RAPTOR (Fig. 3B and C; Supplementary Fig. S3E and S3F). This indicates that high DDX1 expression, as observed in the context of *DDX1*-*MYCN* coamplification, can generate an mTORC1 dependency, serving as a proof of principle.

### DDX1 Influences mTOR Pathway Activity

In the classic dichotomous model of driver and passenger genes, passenger genes are defined as genetic moieties that are altered in their expression or sequence but unlike oncogenes do not drive cancer initiation or progression (21). Although DDX1 has been implicated in many critical cellular activities, such as RNA regulation (22) and DNA damage repair (23), evidence for its oncogenic potential is scarce. To verify the role of DDX1 as a passenger in the pathogenesis of neuroblastoma in an *in vivo* experimental system, we generated a transgenic zebrafish line that stably expresses human DDX1 in the peripheral sympathetic nervous system and compared those to zebrafish expressing both DDX1 and MYCN (Supplementary Fig. S4A–S4C). No tumors developed with transgenic expression of DDX1 alone (Supplementary Fig. S4D), indicating that DDX1 cannot drive tumorigenesis. Neuroblastic tumor penetrance also did not differ between fish expressing MYCN alone (80.9%) or MYCN and DDX1 (79.2%; Supplementary Fig. S4D). In line with our observation in zebrafish, *DDX1-MYCN* coamplification in human neuroblastoma was not associated with differences in overall patient survival compared with patients with tumors only harboring *MYCN* amplifications (Supplementary Fig. S4E). Thus, high levels of DDX1 expression do not significantly alter the initiation or progression of MYCN-driven neuroblastic tumors *in vivo*, confirming that it acts as a *bona fide* passenger gene.

To further test the effect of high DDX1 expression in cancer cells, we selected human neuroblastoma cell lines harboring *MYCN* amplifications, not including *DDX1* and introduced a doxycycline-inducible DDX1 expression vector. Ectopic expression of DDX1 did not affect viability and neuroblastoma cell proliferation (Supplementary Fig. S5A and S5B). In line with its role as a passenger gene, short hairpin RNA (shRNA) mediated DDX1 knockdown in *DDX1*-*MYCN* coamplified neuroblastoma cell lines did not reduce neuroblastoma proliferation (Supplementary Fig. S5C and S5D). Although DDX1 did not influence the tumorigenic properties of neuroblastoma cells, ectopic DDX1 expression was associated with significantly reduced neuroblastoma cell size (Supplementary Fig. S5E and S5F). This suggests that even though DDX1 acts as a *bona fide* passenger gene as per the classic definition, its aberrant expression can influence cellular physiology, which could cause the collateral lethal dependency to mTORC1.

To understand the mechanism of DDX1-induced mTORC1 dependency, we analyzed previously published neuroblastoma gene-expression data from 709 patients (24). Intriguingly, high DDX1 expression was significantly associated with gene-expression programs linked to high mTORC1 pathway activation in primary neuroblastomas (Fig. 3D, q = 0.01, NES = 1.675; Supplementary Table S2; Supplementary Fig. S6A). To test whether DDX1 expression was sufficient to induce mTORC1 pathway activation, we analyzed mTORC1 activity by RNA sequencing and immunoblot analyses of *MYCN*-amplified neuroblastoma cells after ectopic DDX1 expression. Indeed, ectopic DDX1 expression was accompanied by significant differential expression of genes associated with mTORC1 pathway activation (Fig. 3E, q = 0.028, NES = 2.09; Supplementary Table S3 and Supplementary Fig. S6B and S6C). Furthermore, phosphorylation of mTOR at Ser2448 and P70-S6K at Thr389, signs of mTORC1 pathway activation (25, 26), increased in neuroblastoma cells after ectopic DDX1 expression (Fig. 3F; Supplementary Fig. S6D). In turn, shRNA-mediated DDX1 knockdown in *DDX1*-*MYCN* coamplified neuroblastoma cells resulted in reduced phosphorylation of mTOR and P70-S6K (Supplementary Fig. S6E and S6F). This suggests that DDX1 is sufficient to drive mTORC1 pathway activation in the context of *MYCN* amplification without affecting the oncogenic potential of neuroblastoma cells, which could generate the dependency on mTORC1 observed in cancer cells harboring *DDX1-MYCN* amplifications.

### DDX1 Mediates mTORC1 Pathway Activation Through Its Interaction with Alpha-KGDH Complex Members at Its C-terminal Part of a SPRY Domain

**Figure 4.**
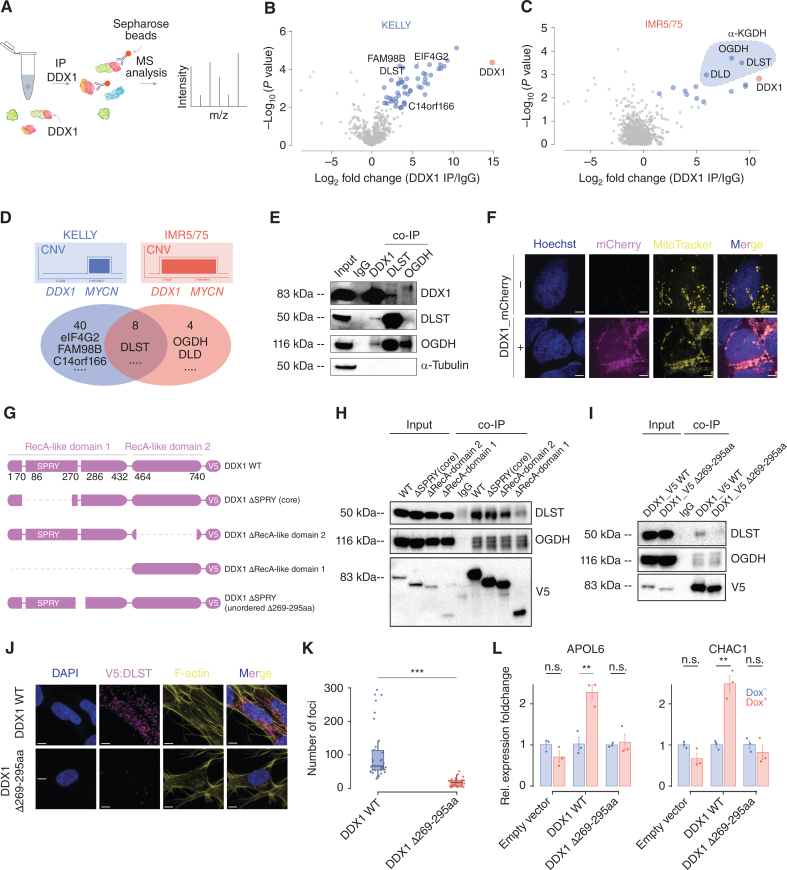
DDX1 interacts with α-KGDH complex members, and its interaction is required for mTORC1 pathway activation. **A,** A schematic of the DDX1 immunoprecipitation (IP) followed by LC-MS/MS. **B** and **C,** Volcano plot of proteins significantly enriched after immunoprecipitation of DDX1 in KELLY cells harboring *MYCN* amplifications without *DDX1* coamplifications (**B**) and in IMR5/75 cells harboring *DDX1-MYCN* coamplifications (**C**) measured using LC-MS/MS (significantly enriched proteins, blue; DDX1 marked in red; dotted line with blue filling marks α-KGDH complex members). **D,** Schematic of the amplicon structure in KELLY and IMR5/75 cells (top). Venn diagram (bottom) of the proteins identified after immunoprecipitation of DDX1 in KELLY cells lacking *DDX1* coamplifications compared with IMR5/75 cells harboring *DDX1-MYCN* coamplifications. **E,** Western immunoblot of DDX1, DLST, OGDH, and α-tubulin in IMR5/75 protein extracts before and after immunoprecipitation using antibodies directed against DDX1, DLST, OGDH, or nonspecific immunoglobulins (IgG). **F,** Representative confocal fluorescence imaging photomicrographs of KELLY cells expressing DDX1-mCherry (magenta), in which mitochondria were stained by MitoTracker DeepRed (yellow) and the nucleus is stained by Hoechst (blue; scale bar, 6 μm). **G,** Schematic illustration of protein domains in DDX1 as well as engineered DDX1 mutants (DDX1-ΔSPRY [core], Δ69-247aa; ΔRecA1, Δ13-472aa; ΔRecA2, Δ493-681aa). **H,** Western immunoblot of V5, DLST, and OGDH before and after immunoprecipitation using antibodies directed against V5, DLST, OGDH, or nonspecific immunoglobulins (IgG) in IMR5/75 cells expressing DDX1-V5 compared with DDX1-ΔSPRY (core), ΔRecA1, or ΔRecA2 truncation mutants, respectively. **I,** Western immunoblot of V5, DLST, and OGDH before and after immunoprecipitation using antibodies directed against V5, DLST, OGDH, or nonspecific immunoglobulins (IgG) in IMR5/75 cells expressing DDX1-V5 or DDX1-V5-Δ269-295aa. **J,** Representative confocal fluorescence imaging photomicrographs of proximity ligation assay signals (magenta dots) in IMR5/75 cells expressing DDX1-V5 or DDX1-V5-Δ269-295aa detected using anti-V5 and anti-DLST antibodies and counterstained with DAPI (blue) and phalloidin (yellow; scale bar, 7 μm). **K,** Quantification of proximity ligation signal (magenta) using anti-DLST and anti-V5 antibodies in IMR5/75 cells expressing DDX1-V5 (*N* = 36) or DDX1-V5-Δ269-295aa (*N* = 34) as shown in **J** (Welch *t* test, *P* = 1.476e−07). **L,** Relative gene expression of mTORC1 downstream pathway genes, as measured by quantitative PCR of KELLY cells inducibly expressing DDX1, DDX1-Δ269-295aa, or an empty vector in the presence and absence of doxycycline (1 μg/mL; Welch *t* test, *P* = 0.125, 0.006, 0.746, 0.064, 0.001, 0.399, respectively; data are shown as mean ± SE; *N* = 3 technical replicates).

To investigate the mechanism by which DDX1 induces mTORC1 pathway activation, we performed immunoprecipitation of DDX1 followed by mass spectrometry–based proteomics in two *MYCN*-amplified neuroblastoma cell lines with and without *DDX1* coamplification to identify proteins that associate with DDX1 in the context of high DDX1 expression, respectively (Fig. 4A–C). In addition to known interactors of DDX1, e.g., eIF4G2, FAM98B, and c14orf166 (27), three members of the α-KGDH complex, DLST, OGDH, and DLD, were significantly enriched after DDX1 immunoprecipitation, particularly in cells with *DDX1-MYCN* coamplification (Fig. 4D; Supplementary Table S4). The interaction of these proteins was confirmed by coimmunoprecipitation followed by immunoblotting (Fig. 4E). The α-KGDH complex predominantly localizes to the mitochondria and critically regulates electron transport chain activity and tricarboxylic acid cycle (TCA) flux. Even though DDX1 is mostly localized in the cytoplasm and nucleus in normal cells, it can associate with mitochondria during embryonal development and immune activation (28). Indeed, ectopically expressed DDX1-mCherry significantly colocalized with thiol-reactive chloromethyl fluorescently labeled mitochondria (Fig. 4F). Thus, DDX1 can interact with α-KGDH complex members in mitochondria.

We next sought to determine the structural basis for the interaction of DDX1 with the α-KGDH complex. DDX ATPases usually contain a structurally conserved core with two RecA-like domains, which catalyze the enzymatic function of DDX proteins. Compared with other DDX ATPases, DDX1 has a unique protein structure. The first RecA-like domain is interrupted by a large SPla and the ryanodine receptor (SPRY) domain (18), which is suspected to function as a protein–protein interaction platform (29). To identify the protein domain required for DDX1:DLST interaction, we generated cells expressing a series of DDX1 domain truncation mutants tagged with V5 (Fig. 4G). Truncation of the entire RecA-like domain 1, including the SPRY domain, strongly and specifically compromised the interaction with DLST, but not OGDH, as evidenced by reduced coimmunoprecipitation (Fig. 4H; Supplementary Fig. S7A). Conversely, no changes in DLST association were observed for a DDX1 truncation mutant lacking the RecA-like domain 2, hinting at the SPRY domain within RecA1 as a possible interaction site with DLST (Fig. 4H; Supplementary Fig. S7A). To test this, we generated a DDX1 mutant lacking the most conserved part of the SPRY domain (AA70-247), which contains the highly conserved surface patch predicted to serve as a protein interaction site (30). Surprisingly, this DDX1 truncation mutant also preserved the interaction with DLST (Fig. 4H; Supplementary Fig. S7A), suggesting that the DDX1:DLST interaction may depend on the less conserved and unordered C-terminal part of the SPRY domain (AA247-295; ref. 30).

Disordered domains of proteins are candidate sites of protein–protein interaction. To test whether a C-terminal part of the SPRY domain may provide the structural scaffold for the interaction with DLST, we searched for intrinsic disordered domains of DDX1, as predicted based on its amino-acid sequences via PONDR (31) and IUPred2A (32). Polynomial modeling of the predicted interaction scores generated by these algorithms nominated amino acids 269aa to 295aa in DDX1 as a candidate disordered domain (Supplementary Fig. S7B). Indeed, immunoprecipitation of the V5-DDX1 Δ269-295aa mutant lacking the predicted disordered domain was associated with reduced DLST coimmunoprecipitation (Fig. 4I). A proximity ligation assay (PLA) with V5-DDX1 Δ269-295aa-expressing cells confirmed the reduced interaction with DLST (Fig. 4J and K). This indicates that the amino-acid stretch 269–295 in the C-terminal part of the SPRY domain of DDX1 is necessary for its interaction with DLST and raises the question of whether this interaction is required for DDX1-mediated mTORC1 activation.

As a key component of the α-KGDH complex, DLST plays an important role in the energy metabolism of cells. Because changes in energy metabolism also directly modulate mTORC1 pathway activation (33), we hypothesized that the interaction of DDX1 with DLST may interfere with α-KGDH function, thereby stimulating mTORC1 activity. To explore the association between mTORC1 pathway activation and the DDX1:DLST interaction, we ectopically overexpressed DDX1 or DDX1 Δ269-295aa in *MYCN*-amplified neuroblastoma cells. DDX1 Δ269-295aa was expressed at similar levels as wild-type DDX1 and localized to mitochondria (Supplementary Fig. S7C and S7D). Ectopic expression of DDX1 but not DDX1 Δ269-295aa was sufficient to increase mTORC1 pathway gene expression (Fig. 4L). Ectopic expression of DDX1 Δ269-295aa was also not associated with increased phosphorylation of P70-S6K at Thr389 in neuroblastoma cells (Supplementary Fig. S7E), suggesting that it was insufficient to induce mTORC1 pathway activation. Thus, DDX1:DLST interaction is required for DDX1-mediated mTORC1 pathway activation.

### DDX1 Alters α-KGDH Complex Activity Resulting in α-KG Accumulation and Reduced Oxidative Phosphorylation (OXPHOS)

**Figure 5.**
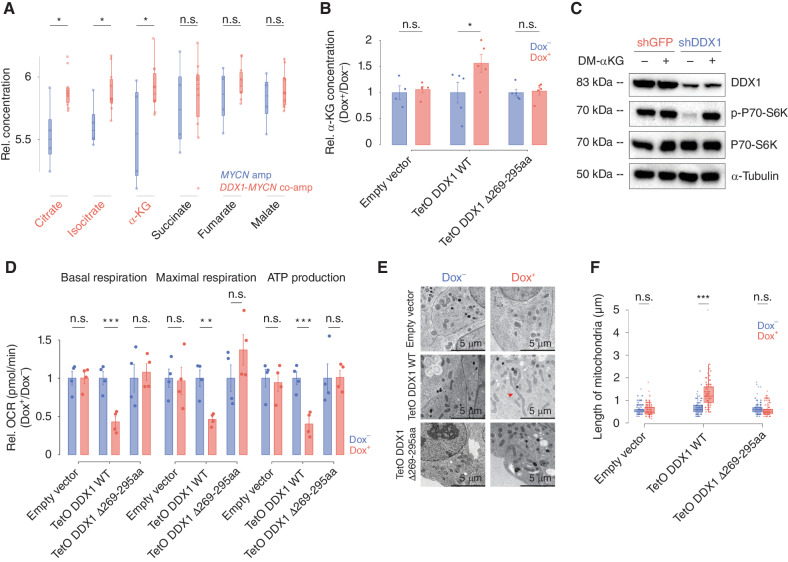
DDX1 hijacks the α-KGDH complex resulting in α-KG accumulation and OXPHOS reduction. **A,** Relative concentrations of α-KG, citrate, and isocitrate in cancer cell lines with *DDX1*-*MYCN* coamplifications (red) compared with cells only harboring *MYCN* amplifications (blue; Welch *t* test, *P* = 0.038764, 0.008224, and 0.025814 for α-KG, citrate, and isocitrate, respectively; *N* = 4 independent *MYCN*-amplified cancer cell lines versus *N* = 8 independent cancer cell lines with *DDX1*-*MYCN* coamplification). **B,** Relative α-KG concentrations measured by GC-MS in KELLY cells ectopically expressing DDX1 or the DDX1-Δ269-295aa for 48 hours. KELLY cells transduced with an empty vector and exposed to doxycycline were used as control (Wilcox test, *P* = 0.02778; data are shown as mean ± standard error). **C**, Western immunoblot of DDX1, P70-S6K, P70-S6K Thr389 phosphorylation, and α-tubulin in IMR5/75 cells treated with DM-αKG (2 mmol/L for 48 hours) and expressing shRNA targeting either DLST or GFP as control. **D,** Mitochondrial oxygen consumption rate (OCR) measured using live-cell metabolic analysis at basal respiration, maximal respiration, and ATP production in KELLY cells inducibly expressing DDX1 or DDX1-Δ269-295aa for 48 hours. KELLY cell transduced with a doxycycline-inducible empty vector served as negative control (Welch *t* test, *P* = 0.002, 0.010, and 0.002 for basal respiration, maximal respiration, and ATP production, respectively; data are shown as mean ± SE; *N* = 4 independent replicates). **E,** Exemplary photomicrographs taken on a transmission electron microscope of cells expressing DDX1 compared with cells expressing DDX1 Δ269-295aa. Cells transduced with an empty vector well as cells not treated with doxycycline served as negative controls. **F,** Quantification of mitochondrial length (longest axis in a cross-section) of cells shown in **E** (Wilcox test, *P* = 0.7954, 7.028e−10, and 0.1453, independently).

Considering the importance of DLST for α-KGDH complex activity in the TCA cycle, we hypothesized that the interaction between DDX1 and DLST may alter the catalytic function of α-KGDH and disrupt TCA cycle flux, which could lead to the accumulation of α-KG and subsequently activate mTORC1 to sustain cell survival, as previously reported in different contexts (34). To test this hypothesis, we analyzed metabolomics data from the CCLE database including measurements of 225 metabolite levels in 928 cell lines from more than 20 cancer types (35) and compared metabolite levels between cells with *DDX1-MYCN* coamplification to those only harboring *MYCN* amplification. In line with altered α-KGDH activity, cancer cells with *DDX1-MYCN* coamplification had higher levels of citrate, isocitrate, and α-KG (Fig. 5A; Supplementary Fig. S8A and Supplementary Table S5). Next, we measured metabolites in neuroblastoma cells ectopically expressing DDX1 compared with cells expressing the truncated DDX1 Δ269-295aa variant or cells expressing physiologic levels of DDX1 using gas chromatography–mass spectrometry (GC-MS). Consistent with decreased α-KGDH activity, α-KG was increased after ectopic DDX1 expression but not when expressing DDX1 Δ269-295aa (Fig. 5B). This indicates that aberrant DDX1 expression is sufficient to alter α-KG levels as a direct result of its interaction with the α-KGDH complex member DLST.

As a rate-determining intermediate in the TCA cycle and the central product of glutaminolysis driving anaplerotic reactions in cells, α-KG can alter mTORC1 activity by modulating its RAS-related GTPase activity (36). We hypothesized that DDX1-mediated mTORC1 activation was because of α-KG accumulation. Indeed, incubation of neuroblastoma cell lines in the presence of membrane-permeable Dimethyl 2-oxoglutarate (DM-KG) was accompanied by mTORC1 pathway activation, phenocopying the effects of DDX1 overexpression (Fig. 5C; Supplementary Fig. S8B and S8C). Disruption of α-KGDH is predicted to impair ATP production through OXPHOS in mitochondria. To test this, we measured oxygen consumption rates as a parameter to study mitochondrial function. Ectopic expression of DDX1, but not of the DDX1 Δ269-295aa mutant, was accompanied by reduced ATP production and respiration (Fig. 5D). In line with the altered TCA cycle, mitochondrial length, a sign of nutrient deprivation (37), was significantly increased in cells expressing DDX1 but not when expressing the DDX1 Δ269-295aa mutant (Fig. 5E and F). Thus, the DDX1:DLST interaction in cells expressing high levels of DDX1 can alter α-KGDH complex activity, resulting in α-KG accumulation, reduced OXPHOS, and compensatory activation of mTORC1 pathway, leading to the pronounced genetic dependency of *DDX1-MYCN* coamplified cells on mTORC1.

### Pharmacologic mTORC1 Inhibition Results in Cell Death in Diverse Models of DDX1-MYCN Coamplification *In Vitro* and *In Vivo*

**Figure 6.**
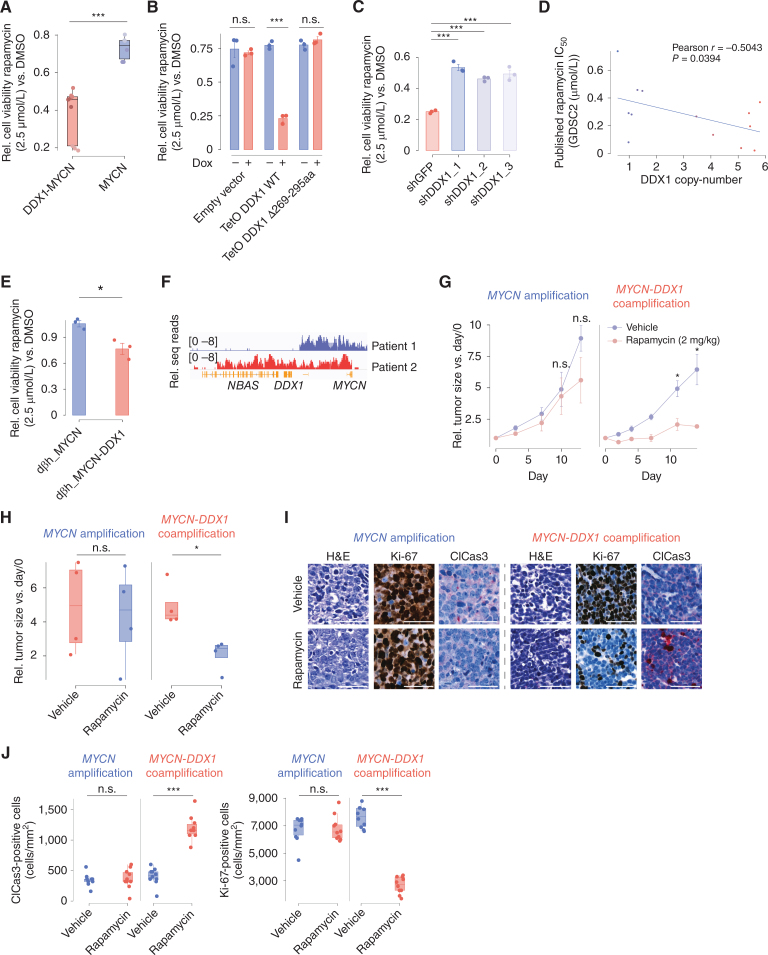
High DDX1 expression is sufficient to increase sensitivity to pharmacologic mTORC1 inhibition *in vitro* and *in vivo*. **A,** Relative cell viability of different neuroblastoma cell lines with *DDX1*-*MYCN* coamplification (red, *N* = 3) or with *MYCN* amplifications (blue, *N* = 2) treated with rapamycin (2.5 μmol/L for 72 hours) compared with cell viability after DMSO (vehicle control) treatment (Welch *t* test, *P* = 2.291e−05 *DDX1*-*MYCN* vs. *MYCN*; each data point represents a technical replicate). **B,** Relative cell viability of KELLY cells inducibly expressing DDX1, DDX1-Δ269-295aa, or an empty vector and treated with rapamycin (2.5 μmol/L for 72 hours) compared with cell viability after DMSO (vehicle control) treatment (Welch *t* test, *P* = 3.943e−05; data are shown as mean ± SE; *N* = 3 technical replicates). **C,** Relative cell viability of IMR5/75 cells expressing shRNAs directed against DDX1 (blue) or GFP (red) and treated with rapamycin (2.5 μmol/L for 72 hours) compared with cell viability after DMSO (vehicle control) treatment (Pairwise *t* test adjusted by Benjamini–Hochberg correction, *P* = 1.3e−05, 4.1e−05, and 2.2e−05 for each independent shRNA directed against DDX1 vs. shGFP, respectively; data are shown as mean ± SE; *N* = 3 technical replicates). **D,** Correlation between the *DDX1* copy number and the IC_50_ value of rapamycin in different neuroblastoma cell lines derived from the GDSC2 database (Pearson correlation, *R* = −0.05043, *P* = 0.0394, *N* = 13 independent cancer cell lines). **E,** Relative cell viability of neuroblastic tumor cells derived from transgenic zebrafish expressing MYCN or MYCN and DDX1 and treated with rapamycin (2.5 μmol/L for 72 hours) compared with cell viability after DMSO (vehicle control) treatment (Welch *t* test, *P* = 0.02707; data are shown as mean ± SE; *N* = 3 independent replicates from cells derived from different zebrafish). **F,** Nanopore sequencing read coverage over the *MYCN* amplicon region in *MYCN-*amplified neuroblastoma PDX with or without *DDX1* coamplification (log-scaled). **G,** Relative change in tumor volume of *MYCN*-amplified NB PDX with or without *DDX1* coamplification treated with rapamycin compared with mice treated with vehicle controls (*N*  =  4 mice per group; *, *P* < 0.05).**H,** Tumor volumes after treatment with rapamycin compared with vehicle treatment in *MYCN*-amplified PDX (*N*  =  4 independent mice per treatment group; *P*  =  0.7918, 0.01286, respectively). **I,** Representative photomicrographs of PDX after IHC staining for cleaved caspase-3 or Ki-67 (scale bar, 50 μm). **J,** Quantification of cleaved caspase-3 or Ki-67-positive cells in PDX shown in **I** (*N*  =  10 sections of 200 μm × 200 μm; *P*  = 0.7454, 1.717e−08, 0.886, and 1.218e−11 independently).

Even though pharmacologic mTOR inhibitors, such as rapamycin, are in clinical use in patients suffering from different cancers, including *MYCN*-amplified neuroblastoma, biomarkers predicting mTOR inhibitor sensitivity are largely lacking. Besides its central role in TCA metabolism, α-KG can broadly influence cellular physiology, for example, as the rate-limiting substrate of 2-oxogluterate-dependent dioxygenases in the management of hypoxia and in epigenetic remodeling (38). Furthermore, the accumulation of α-KG can induce cancer cell differentiation and death (39). Thus, we hypothesized that the α-KG–induced activation of the mTORC1 pathway in cells with *DDX1-MYCN* coamplification was required to sustain cancer cell viability through mTORC1-dependent cell survival mechanisms (40). To test this hypothesis, we incubated *MYCN*-amplified neuroblastoma cell lines with and without *DDX1* coamplification with DM-αKG in the presence and absence of mTORC1 inhibitors. Indeed, cells were more sensitive to DM-αKG–induced cell death when overexpressing DDX1, an effect that was potentiated when combined with the mTORC1 inhibitor rapamycin (Supplementary Fig. S9A–S9D). Moreover, cell lines harboring a *DDX1*-*MYCN* coamplification were more sensitive to rapamycin treatment than cell lines only harboring an *MYCN* amplification (Fig. 6A; Supplementary Fig. S9E). In line with DDX1-induced mTORC1 dependency, ectopic expression of DDX1 increased sensitivity to rapamycin, which was not observed when expressing mutant DDX1 Δ269-295aa (Fig. 6B; Supplementary Fig. S9F), suggesting that the increase in sensitivity depended on the DDX1–DLST interaction. In turn, shRNA-mediated DDX1 knockdown in neuroblastoma cells with a *DDX1*-*MYCN* coamplification resulted in reduced rapamycin sensitivity (Fig. 6C; Supplementary Fig. S9G), indicating that high DDX1 expression was required for mTOR inhibitor sensitivity in the context of *DDX1*-*MYCN* coamplification. Previously published 50% inhibitory concentrations (IC_50_) for rapamycin from the Genomics of Drug Sensitivity in Cancer database (GDSC2; ref. 41) anticorrelated significantly with *DDX1* copy-number in *MYCN*-amplified neuroblastoma cell lines (Fig. 6D, Pearson coefficient = −0.5043 in neuroblastoma cells, *P* = 0.0394), further corroborating the link between *DDX1* coamplification and mTORC1 dependency. Furthermore, DDX1-MYCN expressing neuroblastic zebrafish cells were more sensitive to pharmacologic mTORC1 inhibition than cells only expressing MYCN (Fig. 6E). Next, we sought to test the preclinical antitumor activity of rapamycin *in vivo* in mice harboring patient-derived xenograft models (PDX) of neuroblastomas with *MYCN* amplification. In line with the *in vitro* results, a neuroblastoma PDX model harboring a *DDX1*-*MYCN* coamplification was significantly more sensitive to rapamycin than a model only harboring an *MYCN* amplification, as evidenced by decreased tumor growth and increased histologic signs of cell death, as measured by IHC detection of caspase-3 cleavage (Fig. 6F–J; Supplementary Fig. S9H). In conclusion, high DDX1 expression as a result of *DDX1*-*MYCN* coamplification can induce a therapeutically actionable dependency on mTORC1 *in vitro* and *in vivo*.

## DISCUSSION

With the goal to expand therapeutic strategies in cancer beyond targetable molecular alterations, we found that the coamplification of a passenger gene, which is not directly involved in tumorigenesis, can create pharmacologically actionable amplicon structure–defined collateral lethal therapeutic vulnerabilities. Our analysis of pan-cancer genomes and cancer cell line dependency data further suggests that this strategy may be successful in many cancer entities with diverse oncogene amplifications.

We and others have previously shown that large neighboring genomic regions harboring enhancers coamplify with oncogenes on the same intra- or extrachromosomal DNA amplicon (2, 3). This implies that positive selection acts on these rewired loci. Here, we describe that the coamplification of neighboring genomic regions frequently also results in the inclusion of passenger genes. Under our current model, passenger genes are under neutral selection and represent mere structural bystanders of DNA amplifications. However, it is conceivable that some passenger genes on amplicons provide functional or structural advantages to cancer cells. Functionally, passenger genes could improve tumor cell fitness under special cellular or environmental conditions, for instance, during genome remodeling or altered nutrient supply. Structurally, yet unidentified elements near or within passenger genes may also positively influence amplicon stability, maintenance, or oncogene regulation.

Some new important questions for cancer therapy and treatment resistance directly arise from our observations: First, we observe that many passenger genes are associated with their own, so far uncharacterized therapeutic vulnerabilities. Investigating these collateral vulnerabilities may allow new therapeutic approaches that would substantially improve tumor eradication in high-risk oncogene-driven cancers. Beyond the idea of targeting individual vulnerabilities created by the coamplification of different passenger genes, amplicons also contain a unique chromatin landscape with enhancers required to drive oncogene expression (2, 3). It is tempting to speculate that the structural coupling of genes and their coordinated expression from the joint enhancers could create additional, amplicon-specific vulnerabilities.

The DEAD-Box ATPase DDX1 has previously been implicated in various steps of DNA, mRNA, rRNA, and tRNA processing and repair. Our in-depth investigation of *DDX1* coamplification revealed a previously unanticipated and fundamentally new role of DDX1 in cellular metabolism by uncovering its interaction with the α-KGDH complex as a noncanonical interaction partner of the DLST subunit in neuroblastoma cells. Our data suggest that high DDX1 expression impedes the TCA cycle as well as OXPHOS and consequently promotes accumulation of α-KG, which in turn triggers mTORC1 activation to maintain tumor cell survival. Many nuclear ATPases localize to mitochondria, but to our knowledge, interactions of these ATPases with TCA enzymes or a direct impact on cellular metabolism have not been reported to date. DDX1 is unique amongst other DDX protein family members because it contains a long ∼211 amino-acid insertion between the signature motifs of their ATPase core (18). This insertion encompasses the SPRY core domain and, at its C-terminal end, a relatively unconserved presumably disordered domain, which we found to be essential for the interaction between DDX1 and DLST. This suggests that other DDX protein family members may not be able to interact with TCA enzymes to the same extent as DDX1.

Even though mTORC1 is a well-studied multiprotein complex essential for cancer cell survival, proliferation, and growth (42), biomarkers predicting patient responses to mTOR inhibitors are still largely missing. Intriguingly, rapamycin is part of the treatment protocol RIST (rapamycin, irinotecan, sunitinib, and temozolomide), which was developed for treatment-refractory or relapsed neuroblastomas (43). Even though rapamycin is only one of four agents used in this treatment protocol, it will be important to test whether neuroblastoma patients with *DDX1-MYCN* amplifications respond better to RIST than neuroblastoma patients without such coamplifications. These analyses may reveal that *DDX1-*coamplificaiton could serve as a predictive response biomarker for mTORC1 inhibitor treatment.

In conclusion, we here present a strategy to eliminate cancer cells by targeting factors not directly linked to cancer pathogenesis. In a proof-of-principle study, we investigated a collateral vulnerability in neuroblastoma cells, which is created through passenger gene-mediated metabolic reprogramming. We propose that pharmacologic mTORC1 inhibition could provide an effective therapy for a meaningful fraction of cancer patients with *DDX1*-*MYCN* coamplification. Collectively, our results uncover an additional layer of complexity in DNA amplification, which may provide therapeutic avenues especially in cancers with amplification of oncogenes that have been considered undruggable to date.

## METHODS

### Cell Culture

Human tumor cell lines were obtained from the ATCC or a gift from collaborative laboratories. The identity of all cell lines was verified by short tandem repeat genotyping (Eurofins Genomics). The absence of *Mycoplasma* sp. contamination was determined using a Lonza MycoAlert system (Lonza). Neuroblastoma cell lines were cultured in RPMI-1640 medium (Gibco) supplemented with 1% of penicillin, streptomycin, and 10% of fetal calf serum (FCS; Thermo Fisher). RPE cells were cultured in DMEM (Gibco) supplemented with 1% of penicillin and streptomycin and 10% of FCS. To assess the number of viable cells, cells were trypsinized (Thermo Fisher), resuspended in medium, and sedimented at 300 × *g* for 5 minutes. Cells were then resuspended in medium, mixed in a 1:1 ratio with 0.02% trypan blue (Thermo Fisher), and counted with a Bio-Rad TC20 cell counter.

### Plasmid Constructs

Human *DDX1* cDNA (NM_004939.2) was PCR amplified and isolated from pRecLV151-DDX1 (GeneCopoeia). DDX1 cDNA was cloned into pENTR1A (Thermo Fisher) using restriction enzymes SalI and NotI (New England Biolabs) and cloned into a pInducer20 (Addgene) using the Gateway strategy and the manufacturer's protocol (Thermo Fisher). DDX1 cDNA was cloned into the pRNTR1A vector in frame with C-terminal V5 tag or mCherry and used to generate pInducer20-DDX1-V5 or pInducer20-DDX1-mCherry using the Gateway cloning, according to the manufacturer's instructions. Large truncation of V5-tagged DDX1 lentiviral vectors were generated using site-directed mutagenesis according to the manufacturer's instructions (Q5 Site-Directed Mutagenesis, New England Biolabs) and were confirmed using sanger sequencing. Truncation of core SPRY domain in DDX1: Truncation of the unordered region of the SPRY domain in DDX1: K69-F247 is missing. Truncation of RecA-like domain 1 in DDX1: I13-K472 is missing. Truncation of RecA-like domain 2 in DDX1: K493-V681 is missing. Human *DLST* cDNA (NM_001933.4) was PCR amplified and isolated from a human retro-synthesized cDNA library. DLST cDNA was cloned into pENTR1A using restriction enzymes SalI and NotI (New England Biolabs) and cloned into a pInducer20 using the Gateway strategy and the manufacturer's protocol (Thermo Fisher). pLKO.1 shRNA plasmids targeting DDX1 (TRCN0000050500, TRCN0000050501, and TRCN0000050502), DLST (TRCN0000035426 and TRCN0000035427), and control targeting GFP (shGFP) were obtained from the RNAi Consortium (Broad Institute).

### Lentivirus Production and Cell Transduction

Lentivirus production was carried out as previously described (44). In brief, HEK293T cells were transfected with TransIT-LT1 (Mirus) in a 2:1:1 ratio of the lentiviral vector and psPAX2 and pMD2.G packaging plasmids (Addgene), according to the manufacturer's instructions. Viral supernatant was collected 48 and 72 hours after transfection. The supernatant was pooled, filtered, and stored at –80°C. Neuroblastoma and RPE cells were transduced with virus particles in the presence of 8 μg/mL hexadimethrine bromide (Merck). Cells were transduced for 1 day in antibiotic-free medium and then grown in the full medium for 1 day. Neuroblastoma cells were then selected for 2 days with puromycin hydrochloride (2 μg/mL) or geneticin disulfate (G418, Roth; 2 mg/mL).

### Copy-Number Analysis

The patients’ copy-number data set of Pan-Cancer Analysis of Whole Genomes (PCAWG) study and Tumor Alteration Relevant for Genomics-driven Therapy (TARGET) was retrieved from the cBioPortal database (https://www.cbioportal.org). The high-level amplified genes were labeled as “2” from the profile description. In copy-number data for 556 neuroblastoma patients, cutoffs were chosen to maximize discrimination between *MYCN*-amplified and nonamplified samples (or *DDX1*-amplified and nonamplified samples) and exclude high-level gains: 1.5 for Affymetrix and NimbleGen arrays, 2 for Agilent arrays, and 0.7 for Illumina arrays. Finally, segments with a log2 ratio lower than −2 were called homozygous deletion.

### Dependency Map (DepMap) Data Analysis

CRISPR dependency data (13, bioRxiv 2019.06.31.720243; CERES scores) and gene-level copy-number data (13) were downloaded from the Public Achilles 2021Q1 DepMap release using the Broad Institute's DepMap portal. Cell lines were characterized as being “*DDX1-MYCN* coamplified” if they had *DDX1* and *MYCN* copy-number values that both were greater than or equal to 2 or “*MYCN*-amplified alone” if they had *MYCN* copy-number values that both were greater than or equal to 2 but *DDX1* copy-number value that was less than 2; cell lines with no copy-number data for *DDX1* and *MYCN* were removed from the analysis. From a total cell line in the dependency data set, 12 were classified as *DDX1-MYCN* coamplified, and 8 were classified as *MYCN*-amplified. The Wilcoxon rank-sum test was used to compare dependency scores for each gene between the 2 groups. In Fig. 3B, difference in median gene depletion was plotted on the *x*-axis versus the nominal *P* value of the difference on the *y*-axis. Nominal *P* values are provided. Results of the analysis can be found in a tabular format in the source data.

### Zebrafish Maintenance

Zebrafish (*Danio rerio*) were raised and maintained according to standard protocols at 28°C with a 14/10 hour light-dark cycle (Westerfield, 2000). The transgenic line Tg (*dβh*-*MYCN*: *dβh*-*eGFP*; ref. 45) was a kind gift from Thomas Look (Dana Faber Cancer Institute, Boston, USA). All zebrafish were of the AB background strain. All experiments were performed in accord with the legal authorities approved license “G 0325/19.”

### Zebrafish Transgenesis

Zebrafish line Tg (*dβh*-*MYCN*:*dβh*-*eGFP*) was a kind gift of Thomas Look (Dana-Farber Cancer Institute, Boston, USA) and described previously (45). Plasmid *dβh-eGFP (pDest_IsceI)* was also a kind gift of Thomas Look. Plasmid of Tol2 constructs were a kind gift from Jan-Philipp Junker (Max Delbrück Center, Berlin, Germany). To create *dβh-DDX1-polyA-Tol2-CryAA:mCerulean*, the *dβh* promoter was excised using restriction enzymes ClaI and KpnI (New England Biolabs) and cloned in p5E-MCS (Multiple Cloning Sites, Addgene #26029), linearized with the same enzymes, through T4 Rapid DNA Ligation Kit (Roche). DDX1 was PCR amplified using primers containing suitable recombination sites for the Gateway System (FW: GGGGACAAGTTTGTACAAAAAAGCAGGCTTACCATGGCGGCCTTCT, RV: GGGGACCACTTTGTACAAGAAAGCTGGGTTCTAGAAGGTTCTGAACAGCTGGTTAGG). DDX1 fragment was cloned into pDONR221 (Thermo Fisher) using the Gateway System following the manufacturer's protocol (Thermo Fisher). P3E-polyA and pDEST-Tol2- *CryAA-mCerulean* were a kind gift of Jan-Philipp Junker. P5E-*dβh*, pDONR-DDX1, and p3E-polyA were cloned in a pDEST-Tol2-*CryAA*-*mCerulean* using the Gateway System following the manufacturer's protocol (Thermo Fisher). The final construct was sequenced by LightRun Sequencing (Eurofins Genomics) to confirm the successful reaction. To generate the zebrafish transgenic line Tg (*dβh*-*DDX1*:*CryAA*-*mCerulean*), plasmid *dβh*-DDX1-polyA-Tol2-*CryAA*:*mCerulean* was injected into fertilized eggs, and fish were grown to adulthood.

### Western Immunoblotting

Whole-cell protein lysates were prepared by lysing cells in 15 mmol/L HEPES, 150 mmol/L NaCl, 10 mmol/L EGTA, and 2% (v/v) Triton X-100 supplemented with cOmplete (Roche) and PhosStop (Roche) phosphatase inhibitors. Protein concentrations were assessed by bicinchoninic acid assay (BCA, Santa Cruz Biotechnology). For 5 minutes, 10 μg of protein was denatured in Laemmli buffer at 90°C. Samples were run on NuPage 10% polyacrylamide, 1 mm Tris-Glycine Protein Gels (Thermo Fisher Scientific), and transferred to PVDF membranes (Roche). Membranes were blocked with 5% dry milk or 5% BSA (Roth) in TBS with 0.1% (v/v) Tween-20 (Carl Roth). Membranes were probed with primary antibodies overnight at 4°C and then with secondary antibodies conjugated to horseradish peroxidase for 1 hour at room temperature. Chemiluminescent detection of proteins was carried out using Immunocruz Western blotting luminol reagent (Santa Cruz Biotechnology) and the Fusion FX7 imaging system (Vilber Lourmat). Densitometry was performed using ImageJ (NIH).

### Clonogenic Assay

5,000 cells were seeded in a 24-well plate coated with poly-l-lysine (Merck). After 24 hours, drugs and doxycycline (1 μg/mL) were added to the medium, and fresh medium with drugs and doxycycline was replaced every 48 hours. Cells were continuously cultured for 10 days until the formation of colonies was observed. Cells were fixed with 3.7% formaldehyde for 10 minutes at room temperature, dried, and stained with 0.1% crystal violet (Merck) in 10% ethanol (Roth) for 10 minutes. After washing with sterile water and drying, colonies were measured by ColonyArea (46) from ImageJ (47).

### RNA Sequencing

mRNA was isolated from DDX1-inducibly expressed KELLY cells after 48 hours of incubation in the presence or absence of doxycycline (1 μg/mL, Sigma-Aldrich). Libraries were sequenced on HiSeq 2000 v4 instruments with 2 × 125-bp paired-end reads (Illumina). Reads were mapped with STAR(v2.7.6a) to the human reference genome hg19 with the Gencode v19 annotation using default parameters (48). Gene abundance was estimated using RSEM (v1.3.1; ref. 49), counting only alignments with both mates mapped and allowing for fractional counting of multimapping and multioverlapping reads.

### Mass Spectrometry–Based Proteomics

Beads from immunoprecipitation experiments were resuspended in 20 mL denaturation buffer (6 M Urea, 2 M Thiourea, 10 mmol/L HEPES, pH 8.0), reduced for 30 minutes at 25°C in 12 mmol/L dithiothreitol, followed by alkylation with 40 mmol/L chloroacetamide for 20 minutes at 25°C. Samples were first digested with 0.5 μg endopeptidase LysC (Wako) for 4 hours. After diluting the samples with 80 μL 50 mmol/L ammonium bicarbonate (pH 8.5), 1 μg sequence grade trypsin (Promega) was added overnight at 25°C. The peptide-containing supernatant was collected and acidified with formic acid (1% final concentration) to stop the digestion. Peptides were desalted and cleaned up using Stage Tip protocol (50). After elution with 80% acetonitrile/0.1% formic acid, samples were dried using speedvac, resolved in 3% acetonitrile/0.1% formic acid, and analyzed by LC-MS/MS.

Peptides were separated on a reversed-phase column (20-cm fritless silica microcolumns with an inner diameter of 75 μm), packed with ReproSil-Pur C18-AQ 1.9 μm resin (Dr. Maisch GmbH) using a 90-minute gradient with a 250 nL/minute flow rate of increasing Buffer B concentration (from 2% to 60%) on a High-Performance Liquid Chromatography (HPLC) system (Thermo Fisher), ionized using an electrospray ionization (ESI) source (Thermo Fisher), and analyzed on a Thermo Q Exactive HF-X instrument. The instrument was run in data-dependent mode selecting the top 20 most intense ions in the MS full scans, selecting ions from 350 to 2000 m/z, using 60 K resolution with a 3 × 10^6^ ion count target and 10 ms injection time. Tandem MS was performed at a resolution of 15 K. The MS2 ion count target was set to 1 × 10^5^ with a maximum injection time of 22 ms. Only precursors with charge states 2–6 were selected for MS2. The dynamic exclusion duration was set to 30 seconds with a 10-ppm tolerance around the selected precursor and its isotopes.

Raw data were analyzed using the MaxQuant software package (v1.6.3.4). The internal Andromeda search engine was used to search MS2 spectra against a human UniProt database (HUMAN.2019-07) containing forward and reverse sequences. The search included variable modifications of methionine oxidation, N-terminal acetylation, and fixed modification of carbamidomethyl cysteine. Minimal peptide length was set to 7 amino acids, and a maximum of 3 missed cleavages was allowed. The FDR was set to 1% for peptide and protein identifications. Unique and razor peptides were considered for quantification. Retention times were recalibrated based on the built-in nonlinear time-rescaling algorithm. MS2 identifications were transferred between runs with the “Match between runs” option in which the maximal retention time window was set to 0.7 min. The LFQ (label-free quantitation) algorithm was activated.

The resulting text file was filtered to exclude reverse database hits, potential contaminants, and proteins only identified by site. Statistical data analysis was performed using Perseus software (v1.6.2.1). Log_2_-transformed LFQ intensity values were filtered for a minimum of 3 valid values in at least one experimental group, and missing values were imputed with random low intensity values taken from a normal distribution. Differences in protein abundance between DDX1 bait and IgG control samples were calculated using a two-sample Student *t* test. Proteins enriched in the DDX1 group and passing the significance cutoff (permutation-based FDR < 5%) were defined as DDX1 interactors.

### Gas Chromatography–Mass Spectrometry (GS-MS)

Cells were lysed with 5 mL of ice-cold 50% methanol (MeOH, Honeywell) solution containing 2 μg/mL cinnamic acid (Sigma-Aldrich). Immediately after the MeOH solution was added to the culture plate, lysates were scraped into the MeOH solution and the methanolic lysates were collected. After cell harvest, 4 mL of chloroform (CHCl_3_, VWR), 1.5 mL of MeOH, and 1.5 mL of water (H_2_O, VWR) were added to the methanolic cell extracts, shaken for 60 minutes at 4°C, and centrifuged at 4,149 × *g* for 10 minutes to separate the phases. The polar phase (6 mL) was collected and dried at 30°C at a speed of 1,550 × *g* at 0.1 mbar using a rotational vacuum concentrator (RVC 2-33 CD plus, Christ). Samples were pooled after extraction and used as a quality control (QC) sample to test the technical variability of the instrument. They were prepared alongside the samples in the same way. After drying, samples were split by adding 600 μL of 20% MeOH to the dried extracts, shaking for 60 minutes at 4°C, followed by centrifugation at maximum speed (18,213 × *g*) for 10 minutes. Two 280 μL aliquots per sample were then dried under vacuum, of which one was analyzed and the other kept as a backup.

All polar cell extracts were stored dry at −80°C until analysis. Extracts were removed from the freezer and dried in a rotational vacuum concentrator for 60 minutes before further processing to ensure there was no residual water, which may influence derivatization efficiency. Dried extracts were dissolved in 15 μL of methoxyamine hydrochloride solution (40 mg/mL in pyridine) and incubated for 90 minutes at 30°C with constant shaking, followed by the addition of 50 μL of N-methyl-N-[trimethylsilyl]trifluoroacetamide (MSTFA) including an alkane mixture for retention index determination and incubated at 37°C for 60 minutes. The extracts were centrifuged for 10 minutes at 10,000 × *g*, and aliquots of 25 μL were transferred into glass vials for GC-MS measurement. An identification mixture for reliable compound identification was prepared and derivatized in the same way, and an alkane mixture for reliable retention index calculation was included.

Metabolite analysis was performed on a Pegasus BT GC-TOF-MS-System (LECO Corporation) complemented with an autosampler (Gerstel). Gas chromatographic separation was performed on an Agilent 8890 (Agilent Technologies), equipped with a VF-5 ms column of 30-m length, 250-μm inner diameter, and 0.25-μm film thickness (Agilent Technologies). Helium was used as carrier gas with a 1.2 mL/minute flow rate. Gas chromatography was performed with the following temperature gradient: the first 2 minutes allowed the column to equilibrate at 70°C, and the first temperature gradient was applied at a rate of increase of 5°C per minute until a maximum temperature of 120°C was reached. Subsequently, a second temperature gradient was applied using a rate of increase of 7°C/minute up to a maximum temperature of 200°C. This was immediately followed by a third gradient of 12°C/min up to a maximum temperature of 320°C with a hold time of 7.5 minutes. The spectra were recorded in a mass range of 60 to 600 m/z with a scan rate of 10 spectra/s. A split ratio of 1:5 was used. QC samples were used both for conditioning (6 mouse liver samples at the beginning of the run) the instrument and for measuring technical variability (pooled QC samples) across the batch. The pooled QC samples were run at the beginning and end of each batch and after every 10th sample. The GC-MS chromatograms were processed with the ChromaTOF software (LECO Corporation) including baseline assessment, peak picking, and computation of the area and height of peaks without calibration by using an in-house created reference and a library containing the top 3 masses by intensity for metabolites related to the central carbon metabolism. Data were normalized to the sum of the area. Individual derivatives were summed up. Relative quantities were used. The QC samples were analyzed separately (Supplementary Table S6).

### Coimmunoprecipitation

In the standard coimmunoprecipitation assay, cells were lysed in lysis buffer (50 mmol/L Tris pH 7.5, 150 mmol/L NaCl, 10 mmol/L MgCl_2_, 0.5% Nonidet P40 (Igepal), 10% glycerol, 1 mmol/L NaF, freshly added 1 mmol/L 4-(2-Aminoethyl)benzenesulfonyl fluoride hydrochloride (AEBSF, Sigma-Aldrich), protease inhibitors) and frozen in liquid nitrogen for 1 minute. After thawing at 37°C shortly and removal of cell debris by centrifugation, 1.2 μg of antibody and 100 μL of 20% Protein A-Sepharose beads (Amersham Biosciences) were added to clarified whole-cell extract and incubated overnight at 4°C. The next day, beads then were subjected to three washes with lysis buffer contained 1 mmol/L Dichlorodiphenyltrichloroethane (DTT, Thermo Scientific). The beads were then sent for Mass Spectrometry-based Proteomics of DDX1 interactome or boiled with 1 × Sodium dodecyl sulfate loading buffer at 90°C for western blot analysis. For V5-tagged immunoprecipitation, to assess the binding region of DDX1 to α-KGDH complex, different truncated V5-DDX1s were overexpressed. The same amounts of input and V5 immunoprecipitation eluates were loaded in western blot analysis for the detection of α-KGDH complex.

### Immunofluorescence Staining and Colocalization Analysis

Cells were grown at the desired confluence on a glass cover slide for 24 hours and treated with 1,000 ng/mL doxycycline for another 48 hours (for the corresponding experiment). Cells were washed with phosphate-buffered saline (PBS) three times and fixed for 10 minutes with 4% paraformaldehyde, washed with PBS three times, and permeabilized with PBS containing 0.2% Triton-X100. For immunofluorescence, cells were blocked for 30 minutes in 10% FCS in PBS, incubated overnight at 4°C with the primary antibody, washed three times with PBS-T (0.05% Tween-20 in PBS), incubated for 1 hour in the dark at room temperature with the secondary antibody, washed three times with PBS-T, and mounted on a slide with 4′,6-diamidino-2-phenylindole (DAPI)-containing mounting media. As colocalization staining, DDX1-mCherry or DDX1-mCherry-Δ269-295aa inducibly expressed KELLY cells were seeded on 8-well μ-slide (ibidi) for 48 hours in the presence or absence of doxycycline (1 μg/mL, Sigma-Aldrich). Then, 30 minutes before fixation, cells were incubated with MitoTracker (500 nmol/L, Cell Signaling Technology) and Hoechest (1 μg/mL, Thermo Fisher) at 37°C. After fixation, cells were washed 3 times with PBS and mounted with PBS. Cells were imaged using a Leica TCS SP5 II (Leica Microsystems) and quantified using ImageJ.

### PLA

Cells were seeded into 8-well slides at 3,000 cells per well and treated for 48 hours with doxycycline to induce V5-DDX1 and V5-DDX1(Δ269-295) expression. After fixation for 10 minutes with 4% paraformaldehyde and blocking for 30 minutes with 10% FCS in PBS, cells were incubated overnight at 4°C with primary antibody against V5 (Mouse, Abcam, 1:500) and DLST (Rabbit, Cell Signaling Technology, 1:500). PLA was performed using Duolink *In Situ* Kit (Sigma-Aldrich) according to the manufacturer's protocol. Nuclei were counterstained using Duolink *In Situ* Mounting Medium with DAPI (Sigma-Aldrich) and F-actin stained using phalloidin (Thermo Fisher) according to the manufacturer's instruction. Pictures were taken with a Leica TCS SP5 II (Leica Microsystems) with 63-fold magnification and analyzed using ImageJ.

### RT-qPCR

RNA from cell lines was extracted using an RNeasy mini kit (QIAGEN). Synthesis of cDNA was performed using a Transcription First Strand cDNA Synthesis kit (Roche). Then, 50 ng of cDNA was combined with the corresponding primers (Supplementary Table S7), and SG qPCR Master Mix (Roboklon), keeping the mixture and cycling conditions recommended by the manufacturer. DNA content was measured using a CFX Connect Real-Time PCR detection system (Bio-Rad) with the software CFX Manager (v3.1).

### Oxygen Consumption Rate (OCR) Measurements

The mitochondrial respiratory capacity was determined with the XF Cell Mito Stress Test Kit (Agilent Technologies). Cells were seeded in the XF96 cell culture microplate at a density of 1 × 10^4^ per well with 4 replicates of each condition. XF96 FluxPak sensor cartridge was hydrated with Seahorse Calibrant overnight in a non-CO_2_ incubator at 37°C. The following day, cells were incubated with the Seahorse medium (plus 1 mmol/L pyruvate, 2 mmol/L glutamine, and 10 mmol/L glucose) for 1 hour prior. The OCR was measured by Xfe96 extracellular flux analyzer with the sequential injection of 1 μmol/L oligomycin A, 0.5 μmol/L carbonyl cyanide-p-trifluoromethoxyphenylhydrazon (FCCP), and 0.5 μmol/L rotenone/antimycin A.

### Electron Microscopy

Cells were grown on poly-l-lysine–coated sapphire disks and frozen using the Leica EM ICE. Freeze substitution was done in 1% H_2_O (v/v), 1% glutaraldehyde (v/v), and 1% osmium tetroxide (v/v) in anhydrous acetone using the following protocol: 37 hours at −90°C, 8 hours from −90 to −50°C, 6 hours from −50 to −30°C, 12 hours at −20°C, and 3 hours from −20 to 20°C. Samples were further contrasted with 0.1% uranyl acetate [w/v] in anhydrous acetone and infiltrated with 30%, 70%, and 90% epon-acetone mixtures for 2 hours each, followed by 3 × 2 hour changes of 100% epon (Polybed 812, Science Services) and polymerized at 60°C for 48 hours. Then, 70-nm sections were obtained with an ultra-microtome and imaged at 80 kV with an EM910 (Zeiss) or a Talos L120C (FEI) equipped with a CMOS camera. ImageJ was used for quantification.

### Cell Viability Measurements

Ten thousand cells per well were seeded in transparent, flat-bottom, 96-well plates. After 24 hours, the drug was added to the medium, and cells were incubated for 72 hours. Then, 3-(4,5-dimethylthiazol-2-yl)-2,5-diphenyltetrazolium (MTT) assay reagent (Abcam, ab211091) was added according to the manufacturer's protocol, and the MTT signal was measured by an Epoch plate reader (BioTeK) with read absorbance at OD = 590 nm.

### Zebrafish Tumor Cell Treatment

Tumors from Tg (*dβh*-*MYCN*:*dβh* -*eGFP*; *dβh*-*DDX1*:*CryAA*-*mCerulean*) double transgenic fish and Tg (*dβh*-*MYCN*:*dβh*-*eGFP*) were excised from adult fish immediately after hypothermal shock euthanasia. Tumors were dissociated using a Collagenase II (Thermo Fisher)–based protocol (330 μL Collagenase II—final concentration: 100 U/mL, 120 μL HBSS, 50 μL FCS for 30 minutes at 37°C, followed by 5 minutes of incubation at 37°C after the addition of 200 μL of Dispase II [Thermo Fisher]—final concentration: 2 U/mL, gently pipetting every 10 minutes). After dissociation, single-cell suspension was transferred to Round-Bottom Polystyrene Test Tubes with Cell Strainer Snap Cap to remove undissociated tissue. Cells were resuspended in DMEM (Gibco) supplemented with 10% FCS and 1% penicillin/streptomycin and plated in 96-well plates at a density of 0.1 × 10^5^ cells. Cells were incubated at 27°C, 5% CO_2_. After 24 hours, cells were visually inspected for eGFP expression using an AXIO microscope (Zeiss), and rapamycin (2.5 μmol/L) or vehicle was added to the DMSO; cells were then incubated for 72 hours, and viability was assessed through MTT assay (Abcam) following the manufacturer's protocol. Absorbance was measured using an Epoch plate reader (BioTeK) at OD = 590.

### Nanopore Sequencing

The tumor sample was sliced, and DNA was extracted using the NucleoSpin Tissue kit (Macherey-Nagel GmbH & Co. KG). Libraries were prepared using the ONT Rapid Barcoding Kit (catalog no. SQK-RBK004, Oxford Nanopore Technologies) according to the manufacturer's instructions, and sequenced on an R9.4.1 MinION flowcell (FLO-MIN106, Oxford Nanopore Technologies). A maximum of four samples were multiplexed per run. Nanopore data were base-called and demultiplexed using Guppy (v.5.0.14; running guppy_basecaller with dna_r9.4.1_450bps_hac model and guppy_barcoder with FLO-MIN106 and default parameters). The obtained reads were quality filtered using NanoFilt (v.2.8.0; -l 100–headcrop 50–tailcrop 50) and aligned using ngmlr (v.0.2.7) against the GRCh37/hg19 reference genome. Genome tracks with rad densities (log-scaled) were created by deepTools.

### PDX Treatment

The establishment of PDX models was conducted as previously described in collaboration with Experimental Pharmacology & Oncology GmbH (EPO). Briefly, a tumor fragment was serially transplanted in mice at least three times prior to the experiments. All experiments were conducted according to the institutional animal protocols and the national laws and regulations and approved by the Charité University Medicine. *MYCN* amplification status was determined by florescence *in situ* hybridization (FISH) at the time of diagnosis. *DDX1* coamplification status was determined by Nanopore sequencing. Thus, PDX was classified by *MYCN* amplification and *MYCN-DDX1* coamplification. Tumor fragments from neuroblastoma patients were transplanted subcutaneously into the flanks of NSG-H(NOD.Cg-*Prkdc^scid^ Hprt^em1Mvw^ Il2rg^tm1Wjl^*/*MvwJ*) female mice between 6 and 8 weeks old. Animals were IVC housed under sterile and standardized conditions (22°C ± 1°C, 50% relative humidity, 12-hour light-dark cycle, autoclaved food, bedding material, and tap water ad libitum). Tumor growth was monitored with caliper measurements, and tumor volume was calculated with the formula length × width^2^/2. PDX were serially transplanted in mice at least three times prior to the experiments. On the first day of the treatment, all the transplanted tumors were measured, and they were all with a similar size of around 0.1 cm^3^. Mice were randomized into 2 groups with at least 3 mice to receive Rapamycin (2 mg/kg, intravenously) and vehicle. Rapamycin was dissolved in 4% DMSO and 99.6% Miglyol. Solutions in which the drugs were dissolved were used as vehicle controls. Mice were sacrificed by cervical dislocation once the tumor volume exceeded 2000 mm^3^ or body weight loss was higher than 10%. For IHC staining of cleaved caspase-3 and Ki-67, snap-frozen tumor fragments were cut and stained following the standard protocol using the antibodies listed in Supplementary Table S7.

### Statistical Analysis

All experiments were performed a minimum of three times with a minimum of three independent measurements. All statistical analysis was performed with R 3.6, R 4.0, or Python 3.7. All data are represented as mean ± standard error. Statistical significance was defined as *, *P* < 0.05; **, *P* < 0.01; ***, *P* < 0.001.

### Data Availability

Copy-number data for 556 neuroblastoma patients were downloaded from https://github.com/padpuydt/copynumber_HR_NB/. Public data of 709 neuroblastoma patients’ microarrays supporting the findings of this manuscript were downloaded from ArrayExpress under accession E-MTAB-1781. The cancer cell line metabolism data set was downloaded from DepMap (35). The public drug response data set (GDSC2) was downloaded from https://www.cancerrxgene.org/. CRISPR dependency data (ref. 13; CERES scores) and gene-level copy-number data (13) were downloaded from the Public Achilles 2021Q1 DepMap. Pan-Cancer Analysis of Whole Genomes (PCAWG) study (8) and Tumor Alteration Relevant for Genomics-driven Therapy (TARGET) database (9, 10) from cBioPortal. All other data are available from the corresponding authors upon reasonable request.

### Code Availability

Code is available at https://github.com/yeebae1118/DDX1_Bei.

## Supplementary Material

Supplementary Figures S1-S9 Legends for supplementarytables and supplementary figures. Supplementary Figure S1. Passenger genes are frequently co-amplified with oncogenes in cancers. Supplementary Figure S2. DDX1 is highly expressed when co-amplified with MYCN. Supplementary Figure S3. Neuroblastoma cell lines with DDX1-MYCN co-amplification depend on mTORC1. Supplementary Figure S4. Ectopic DDX1 expression does not alter MYCN-driven tumorigenesis in zebrafish. Supplementary Figure S5.DDX1 expression does not affect tumorigenic properties of cancer cell lines but induces changes in cell size. Supplementary Figure S6. Aberrant DDX1 overexpression results in mTOCR1 pathway activation. Supplementary Figure S7. DDX1 interacts with alpha-KGDH complex members and disruption of the DDX1:DLST interaction reduces mTORC1 pathway activation. Supplementary Figure S8. High DDX1 expression is associated with α-KG accumulation and OXPHOS reduction. Supplementary Figure S9. Aberrant DDX1 expression is associated with increased sensitivity to αKG and pharmacological mTORC1 inhibition.

Supplementary Table S1 List of gene dependencies associated with DDX1 co-amplification.

Supplementary Table S2 Gene sets enriched in in primary neuroblastomas with DDX1 co-amplification.

Supplementary Table S3 Gene sets enriched in in cell lines after ectopic DDX1 expression.

Supplementary Table S4 Peptides significantly enriched after DDX1 immunoprecipitation as measured using LC-MS/MS.

Supplementary Table S5 Metabolite levels in cells with DDX1-MYCN co-amplification compared to cell lines without such co-amplification.

Supplementary Table S6 QC sample reporting for Gas chromatography–mass spectrometry (GS-MS)

Supplementary Table S7 List of antibodies, materials, oligonucleotides, deposited data and software used in this study.
