# Supplementary material for: Passenger Gene Coamplifications Create Collateral Therapeutic Vulnerabilities in Cancer
Source: Cancer Discov. 2024 Jan 13;14(3):492–507. doi: 10.1158/2159-8290.CD-23-1189 (PMC10911929; doi:10.1158/2159-8290.CD-23-1189)
Supplement: Supplementary Figures S1-S9 — Legends for supplementarytables and supplementary figures. Supplementary Figure S1. Passenger genes are frequently co-amplified with oncogenes in cancers. Supplementary Figure S2. DDX1 is highly expressed when co-amplified with MYCN. Supplementary Figure S3. Neuroblastoma cell lines with DDX1-MYCN co-amplification depend on mTORC1. Supplementary Figure S4. Ectopic DDX1 expression does not alter MYCN-driven tumorigenesis in zebrafish. Supplementary Figure S5.DDX1 expression does not affect tumorigenic properties of cancer cell lines but induces changes in cell size. Supplementary Figure S6. Aberrant DDX1 overexpression results in mTOCR1 pathway activation. Supplementary Figure S7. DDX1 interacts with alpha-KGDH complex members and disruption of the DDX1:DLST interaction reduces mTORC1 pathway activation. Supplementary Figure S8. High DDX1 expression is associated with α-KG accumulation and OXPHOS reduction. Supplementary Figure S9. Aberrant DDX1 expression is associated with increased sensitivity to αKG and pharmacological mTORC1 inhibition. [file cd-23-1189_supplementary_figures_s1-s9_suppsf1.docx]

**Supplementary files for: Passenger gene co-amplifications create collateral therapeutic vulnerabilities in cancer**

**Supplementary tables**

Table S1. List of gene dependencies associated with DDX1 co-amplification.

Table S2. Gene sets enriched in in primary neuroblastomas with DDX1 co-amplification.

Table S3. Gene sets enriched in in cell lines after ectopic DDX1 expression.

Table S4. Peptides significantly enriched after DDX1 immunoprecipitation as measured using LC-MS/MS.

Table S5. Metabolite levels in cells with DDX1-MYCN co-amplification compared to cell lines without such co-amplification.

Table S6. QC sample reporting for Gas chromatography–mass spectrometry (GS-MS)

Table S7. List of antibodies, materials, oligonucleotides, deposited data and software used in this study.

**Supplementary figures**

**
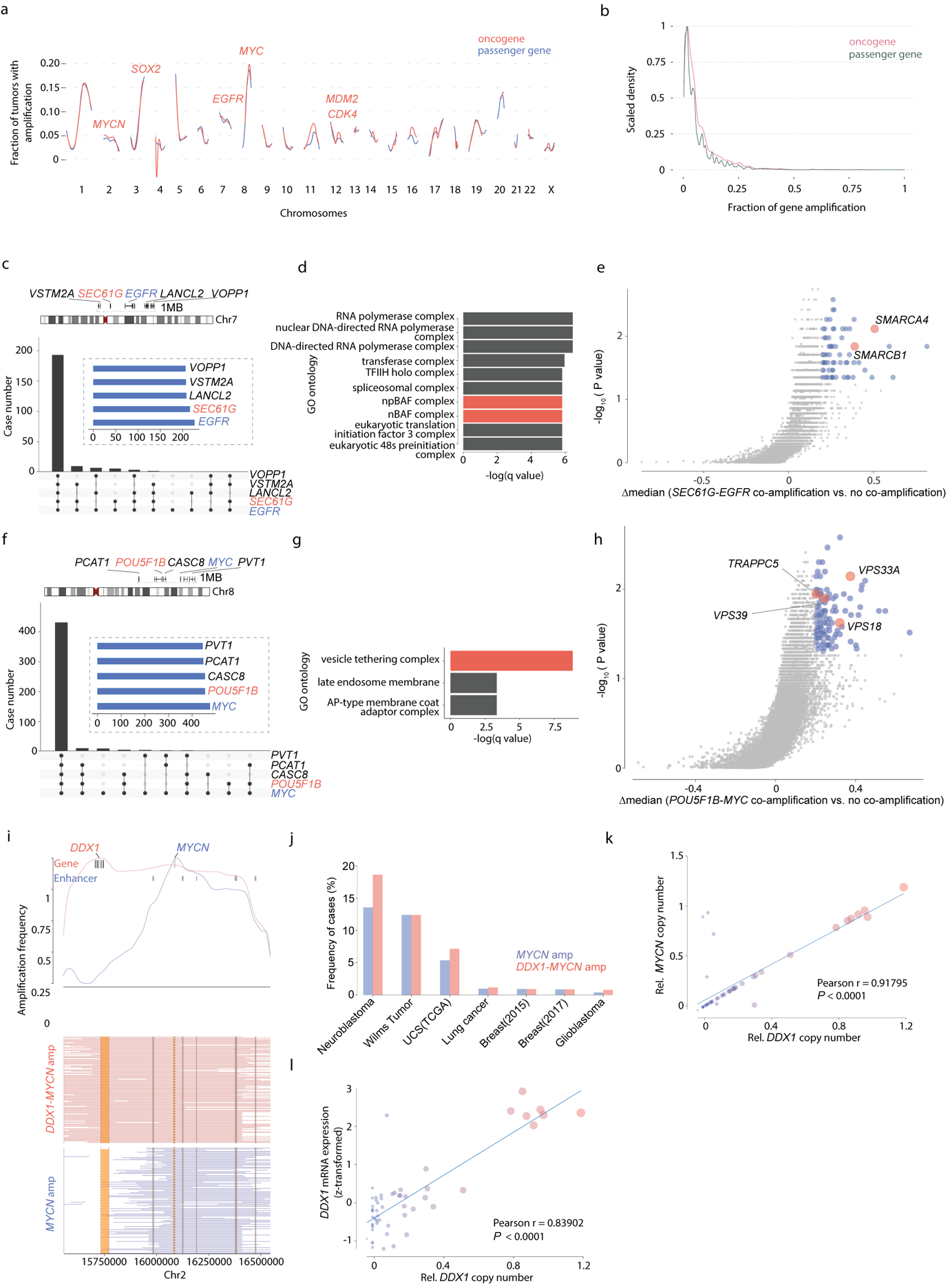
**

**Supplementary Figure S1. Passenger genes are frequently co-amplified with oncogenes in cancers. a,** Fraction of tumors (*N* = 2970) from PCAWG and TARGET datasets with oncogene amplifications (red) or passenger gene amplifications (blue) throughout the genome fitted by local regression (LOESS). The annotated genes are amongst the most commonly altered oncogenes in cancers. **b,** Histogram of the fraction of tumors with oncogene and passenger gene amplification in each tumor entity (*N* = 2970 total number of cancer patients). **c,** Chromosome 7 schematic highlighting the area of *EGFR* amplification and passenger genes recurrently included on the amplicon (top). Upset plot (bottom) for the co-amplification frequency of three passenger genes, *VOPP1*, *VSTM2A*, LANCL2 and *SEC61G*, identified on the *EGFR* amplicon in a cohort of PCAWG. **d**, Top 10 GO ontology terms enriched for collateral lethal targets associated with *SEC61G*-*EGFR* co-amplifications (nBAF complex labeled in red). **e**, Difference in gene dependency scores between cancer cell lines with *SEC61G* -*EGFR* co-amplification vs. cell lines with *EGFR* amplifications compared to the log-transformed *P* values (Wilcoxon; candidate collateral lethal dependencies in *SEC61G* -*EGFR* co-amplified cancer cells, blue; nBAF complex, red). **f**, Chromosome 8 schematic highlighting the area of *MYC* amplification and passenger genes recurrently included on the amplicon (top). Upset plot (bottom) for the co-amplification frequency of three passenger genes, *PVT1*, *PCAT1, CASC8* and *POU5F1B*, identified on the *MYC* amplicon in a cohort of PCAWG. **g**, Significant top 3 GO ontology terms enriched for collateral lethal targets associated with *POU5F1B* -*MYC* co-amplifications (vesicle tethering complex labeled in red). **h**, Difference in gene dependency scores between cancer cell lines with *POU5F1B* -*MYC* co-amplification vs. cell lines with *MYC* amplifications compared to the log-transformed *P* values (Wilcoxon; candidate collateral lethal dependencies in *POU5F1B* -*MYC* co-amplified cancer cells, blue; vesicle tethering complex highlighted in red). **i,** Density plot for the amplification frequency near *MYCN,* measured using copy number profiles from *238* *MYCN*-amplified neuroblastoma patients with (red) and without (blue) *DDX1* co-amplification. **j,** Frequency of *MYCN* amplifications with (red) and without (blue) *DDX1* co-amplification in different tumor entities (UCS, uterine carcinosarcoma; Lung cancer, lung adenocarcinoma. Breast 2015, BRCA_igr_2015; Breast 2017, BRCA_mbcproject_wagle_2017). **k,** Correlation between *DDX1* copy number and *MYCN* copy number derived from the TARGET neuroblastoma dataset (Pearson r = 0.91795, *P* < 0.0001, *N* = 59). Size of dots reflects the relative *MYCN* and *DDX1* copy number. **l,** Correlation between *DDX1* copy number and DDX1 mRNA expression derived from the TARGET neuroblastoma dataset (microarray) (Pearson r = 0.83952, *P* < 0.0001, *N* = 59).


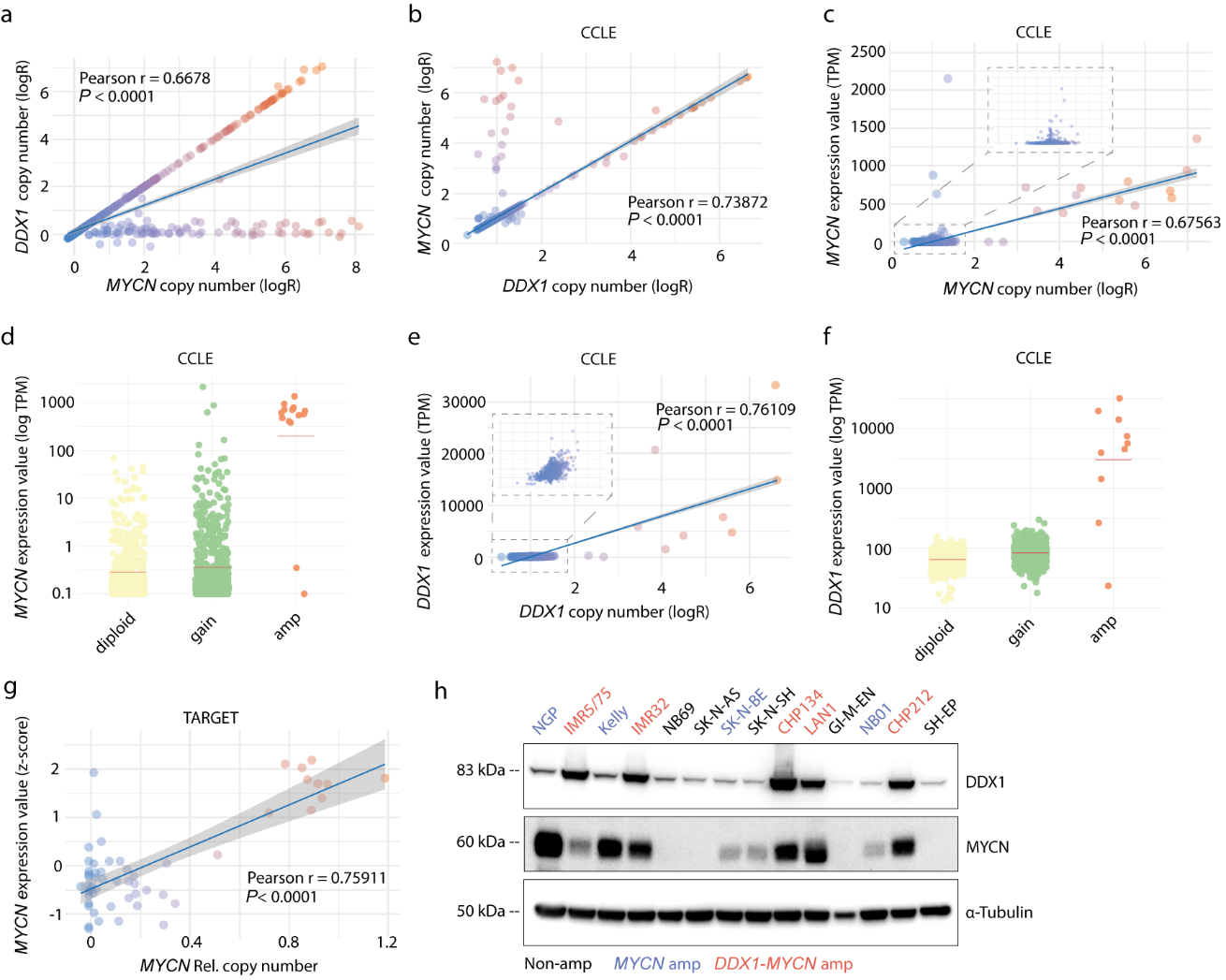


**Supplementary Figure S2. *DDX1* is highly expressed when co-amplified with *MYCN*.**

**a,** *DDX1* copy number compared to *MYCN* copy number in a cohort of 556-neuroblastomas (*N* =556, Pearson r = 0.6678, *P* < 0.0001, *N* = 556). **b,** *DDX1* copy number compared to *MYCN* copy number in cancer cell lines from the CCLE database (*N* = 1713, Pearson r = 0.73872, *P* < 0.0001). **c,** Correlation between *MYCN* copy number and *MYCN* mRNA expression in cancer cell lines from the CCLE database (*N* = 1020, Pearson r = 0.67563, *P* < 0.0001). **d,** *MYCN* mRNA expression in cancer cell lines from the CCLE database (*MYCN* diploid refers to a copy number logR of 1, *MYCN* gain refers to copy numbers logR >1 and <2 and *MYCN* amplification refers to copy numbers of >2). **e,** Correlation between *DDX1* copy number and *DDX1* mRNA expression in cancer cell lines from the CCLE database (*N* = 1020, Pearson r = 0.76109, P < 0.0001). **f,** *DDX1* mRNA expression in in cancer cell lines from the CCLE database, i.e., *DDX1* diploid vs. *DDX1* gain vs. *DDX1* amplification. **g,** *MYCN* copy number compared to MYCN expression in primary neuroblastomas from the TARGET dataset (*N* = 59, Pearson r = 0.75911, P < 0.0001,). **h,** Western immunoblot of DDX1 and MYCN in a panel of neuroblastoma cell lines with (red) and without (blue) *DDX1-MYCN* co-amplifications, compared to cell lines without *MYCN* amplifications (black).


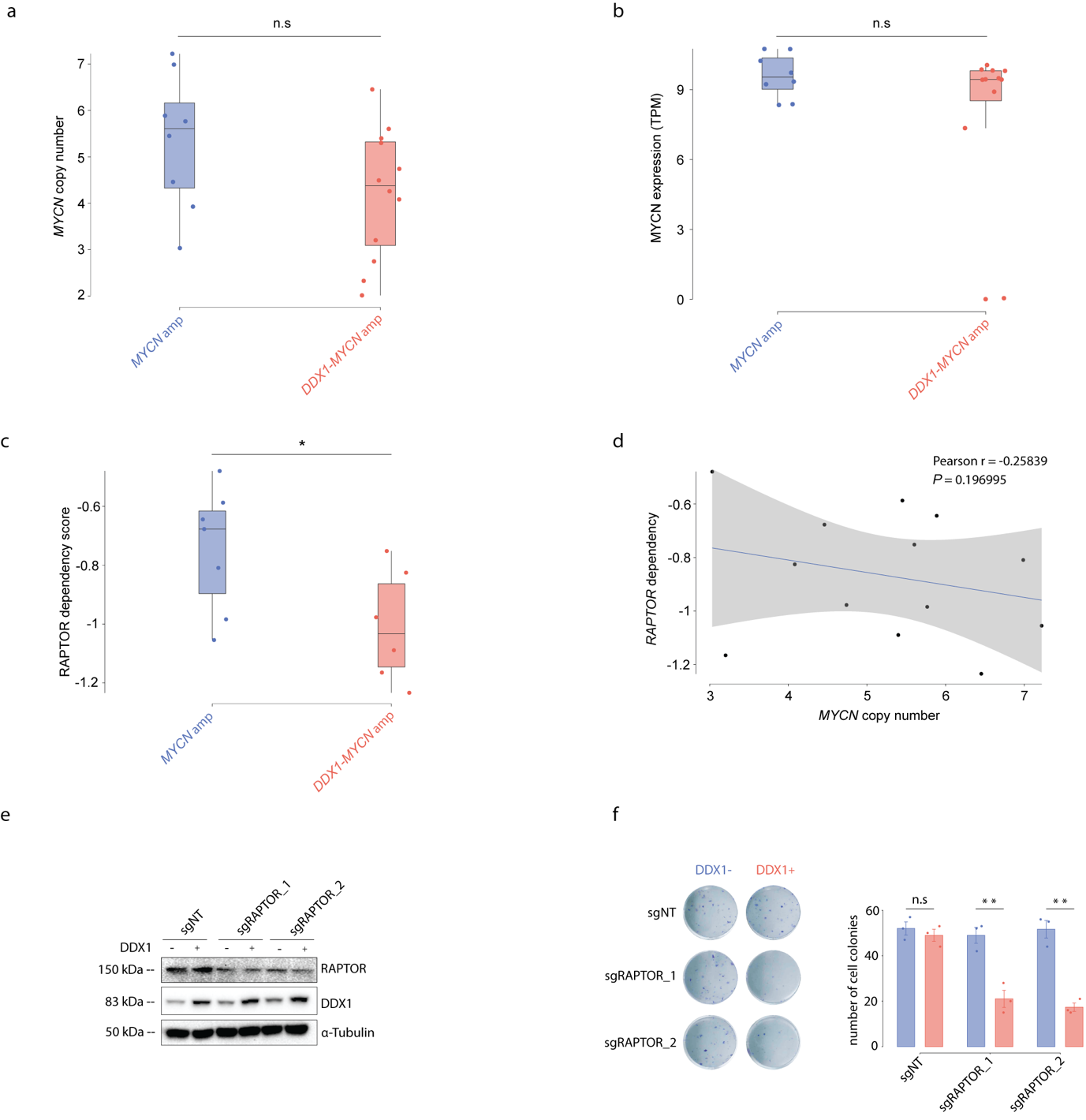


**Supplementary Figure S3. Neuroblastoma cell lines with *DDX1-MYCN* co-amplification depend on mTORC1. a**, Boxplot presenting the distribution of *MYCN* copy number between *MYCN* amplified (*N* = 8) and *DDX1-MYCN* co-amplified (*N* = 12) cancer cell lines**.** Statistical analysis was performed by Wilcox test (*P* = 0.1153). **b,** Boxplot presenting the distribution of MYCN expression between *MYCN* amplified (*N* = 8) and *DDX1-MYCN* co-amplified (*N* = 12) cancer cell lines**.** Statistical analysis was performed by Wilcox test (*P* = 0.4727). **c,** Boxplot presenting the distribution of *RAPTOR* dependency score between *MYCN* amplified (*N* = 7) and *DDX1-MYCN* co-amplified (*N* = 6) neuroblastoma cell lines**.** Statistical analysis was performed by Wilcox test (*P* = 0.02564). **d,** Correlation between *MYCN* copy number and the CRISPR-based dependency score from DepMap for *RAPTOR* in neuroblastoma cell lines (Pearson correlation analysis, Pearson r = -0.258396, *P* = 0.19699, *N* = 13). **e,** Western immunoblot analysis of RAPTOR in NGP cells after induced DDX1 expression and incomplete RAPTOR knockout by two independent sgRNAs targeting *RAPTOR* compared to a non-targeting sgRNA. **f,** Representative images of cell colonies formed by NGP cells transduced with the doxycycline-inducible DDX1-mcherry vectors and with two pairs of sgRNA targeting *RAPTOR (*sgRAPTOR*)* or non-target sgRNA (sgNT) as well as Cas9 in the presence and absence of doxycycline (1 µg/ml) and stained with crystal violet (left). Quantification of colony numbers (right, mean ± s.e. *N* = 3 biological replicates). (Welch’s t-test, *P* = 0.4866132, 0.005699854 and 0.004625467 for sgNT, sgRAPTOR_1 and sgRAPTOR_2, respectively).


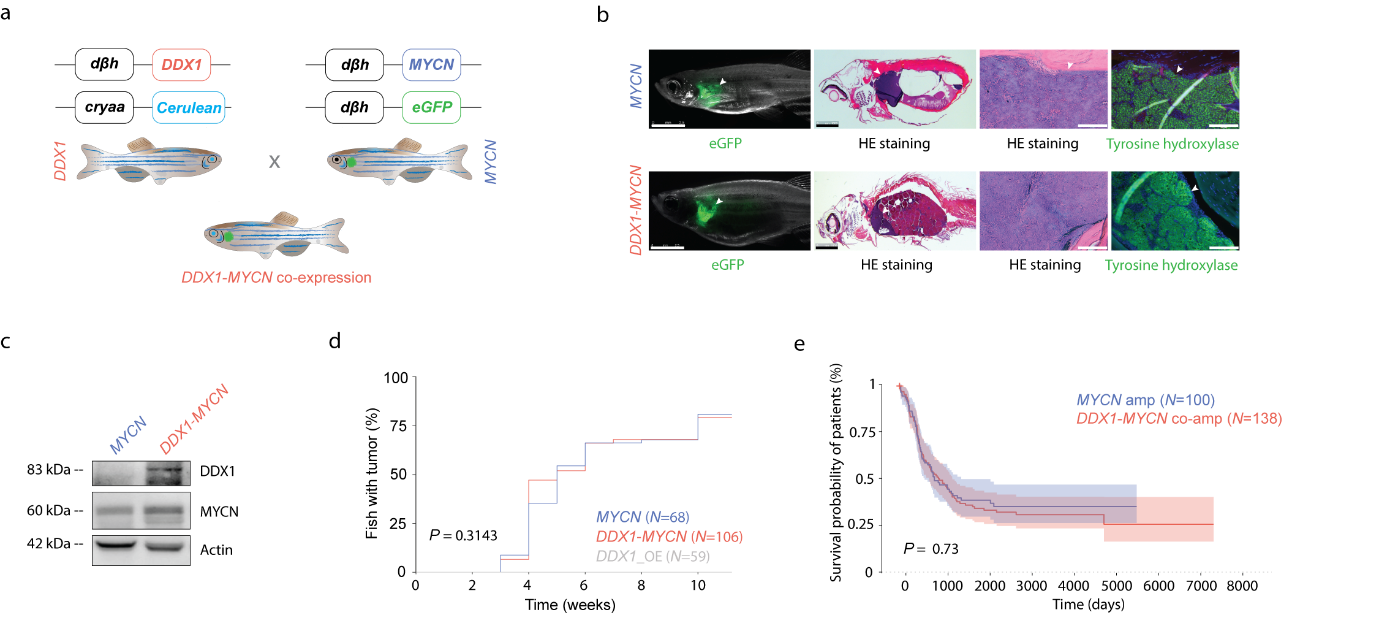


**Supplementary Figure S4. Ectopic DDX1 expression does not alter MYCN-driven tumorigenesis in zebrafish. a,** Schematic figure showing the generation of DDX1-MYCN co-expressing zebrafish through breeding of tg (*dβh*-*DDX1*:*CryAA*-*mCerulean*) and tg (*dβh*-*MYCN*: *dβh*-*eGFP*) zebrafish. **b,** From left to right, exemplary images from transgenic zebrafish tg (*dβh*-*DDX1*:*CryAA*-*mCerulean*) and tg (*dβh*-*MYCN*: *dβh*-*eGFP*) green fluorescent neuroblastic tumors in the adrenal medulla analogue (interrenal gland, white arrowhead). Hematoxylin & Eosin (HE) staining of sagittal paraffin sections from the same fish. Magnification into the tumor area from sections shown left, with HE and tyrosine hydroxylase (green) staining. Scale bar from left to right: 2.5 mm, 1 mm, 100 µm and 100 µm. Arrowheads point to neuroblastic tumors in zebrafish. **c,** Western immunoblot of DDX1 and MYCN in zebrafish tumor cell extracts. **d,** Cumulative frequency of neuroblastic tumors in stable transgenic zebrafish by Kaplan-Meier analysis (*DDX1-MYCN* vs. *MYCN*, Kolmogorow-Smirnow-Test*, P* = 0.8494). **e,** Kaplan-Meier analysis of patients with *DDX1*-*MYCN* co-amplification compared to patients with *MYCN* amplifications lacking *DDX1* co-amplification (Log-Rank Test, *P* = 0.73).


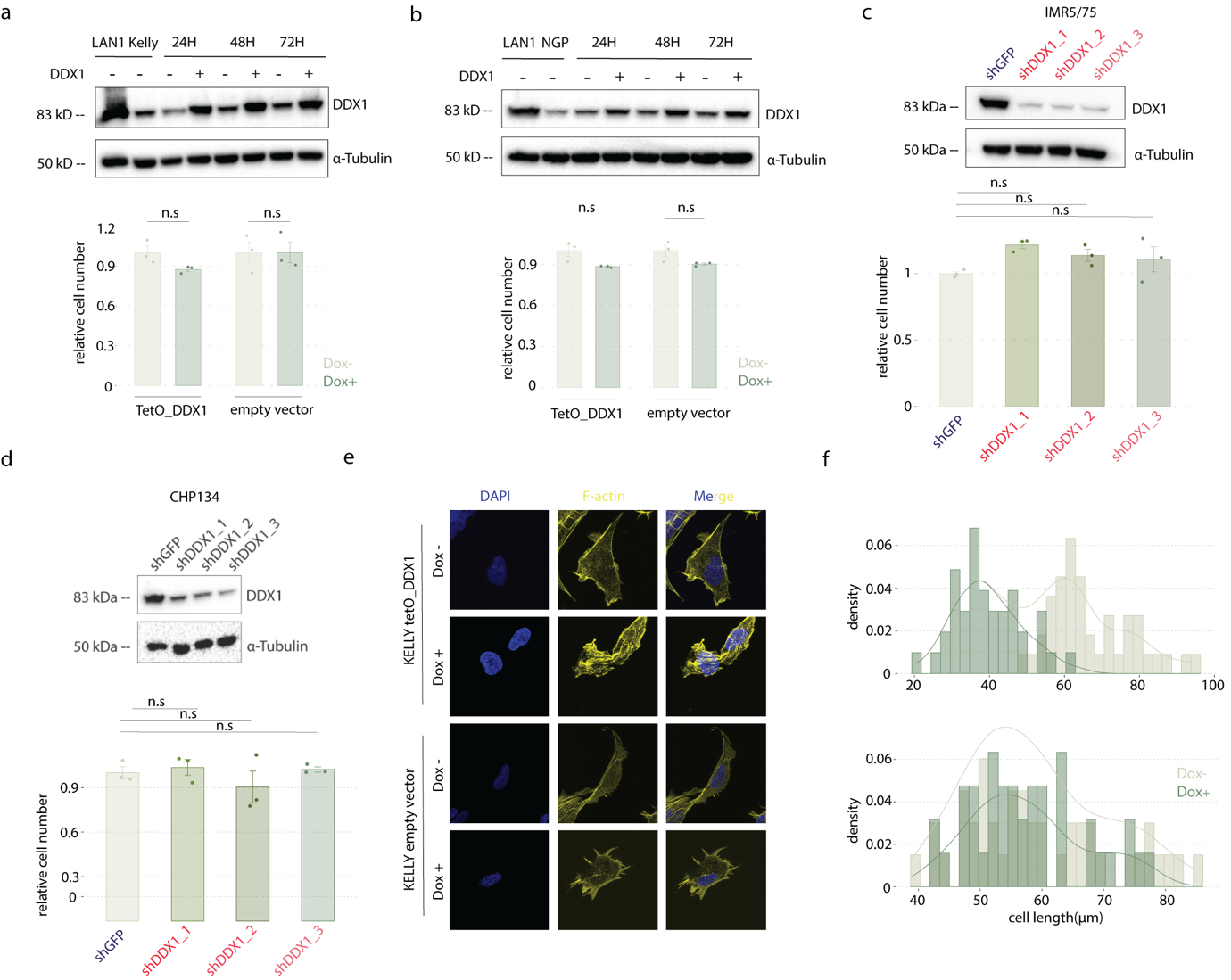


**Supplementary Figure S5. DDX1 expression does not affect tumorigenic properties of cancer cell lines but induces changes in cell size. a,** Western immunoblot of DDX1 in KELLY cell after inducible expression of DDX1 (1000ng/ml doxycycline treatment for 24, 48 and 72 hours) with LAN1 serving as a positive control and α-tubulin as loading control (top). Relative number of viable KELLY cells after inducible expression of DDX1 for 7 days. (bottom, Welch’s t-test, *P* = 0.1175 and 0.9852 for cells transduced with an empty vector and cells with an inducible vector treated with doxycycline or vehicle control, respectively). Data are shown as mean ± s.e. (*N* = 3). KELLY cell transduced with an empty vector served as a negative control. **b,** Western immunoblot of DDX1 expression in NGP cell after inducible expression of DDX1 (1000ng/ml doxycycline treatment for 24, 48 and 72 hours) with LAN1 serving as a positive control and α-tubulin as loading control (top). Relative number of viable NGP cells after inducible expression of DDX1 for 7 days. (bottom, Welch’s t-test, *P* = 0.1061 and 0.1472 for cells transduced with an empty vector and cells with an inducible vector treated with doxycycline or vehicle control, respectively). Data are shown as mean ± s.e. (*N* = 3). NGP cells transduced with an empty vector served as a negative control. **c,** Western immunoblot of DDX1 in IMR5/75 cells transduced to express shRNAs targeting DDX1 as well as an shRNA targeting GFP serving as a negative control (top). Relative number of viable IMR5/75 cells expressing shRNA targeting DDX1 for 7 days compared to cells expressing a shRNA targeting GFP. (bottom, Pairwise t-test adjusted by Benjamini-Hochberg correction, *P* = 0.14, 0.31 and 0.31 for all shRNAs, respectively). Data are shown as mean ± s.e. (*N* = 3). **d,** Western immunoblot of DDX1 in CHP134 cells expressing shRNAs targeting DDX1 as well as an shRNA targeting GFP serving as a negative control (top). Relative number of CHP134 cells expressing shRNAs targeting DDX1 for 7 days compared to cells expressing a shRNA targeting GFP. (bottom, pairwise t-test adjusted by Benjamini-Hochberg correction, *P* = 0.88, 0.64 and 0.88 for all shRNAs, respectively). Data are shown as mean ± s.e. (*N* = 3). **e,** Representative immunofluorescence images of KELLY cells after inducible expression of DDX1 (1000ng/ml doxycycline treatment for 48 hours). Nucleus and actin cytoskeleton were stained with DAPI (blue) and phalloidin (yellow), respectively. Scale bar: 12 µm. KELLY cells transduced with an empty vector served as negative control. **f**, Histogram of the cellular length of KELLY cells after inducible expression of DDX1 (1 µg/ml doxycycline treatment for 48 hours). KELLY cells transduced with an empty vector served as negative control.


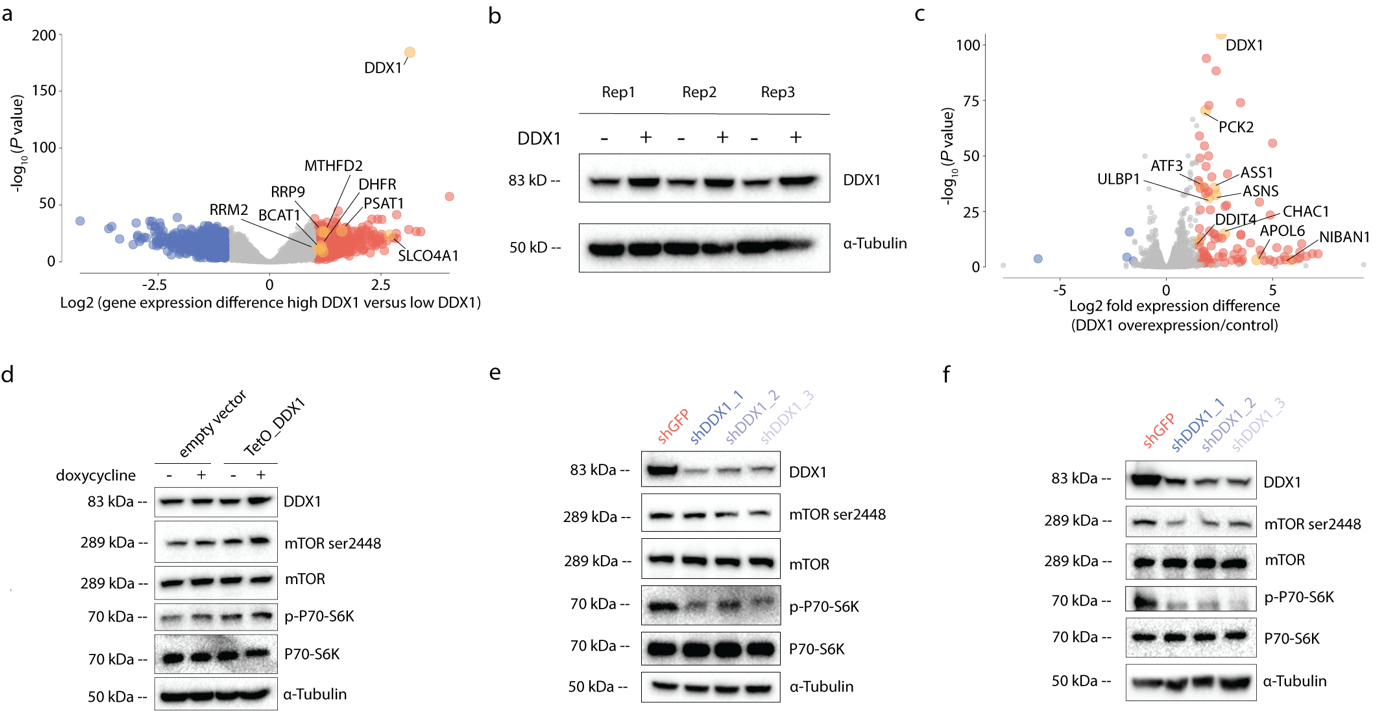


**Supplementary Figure S6. Aberrant DDX1 overexpression results in mTOCR1 pathway activation**. **a,** Volcano plot of genes differentially expressed between primary neuroblastomas with high vs. low *DDX1* mRNA expression (*N* = 709 patients; genes with significantly lower expression, blue; genes with significantly higher expression, red, genes known to be regulated by mTORC1 signaling, orange). **b,** Western immunoblot of DDX1 in KELLY cells after inducible expression of DDX1 (1000ng/ml doxycycline treatment for 48 hours) with three independent biological replicates. **c**, Volcano plot of genes differentially expressed in KELLY cells with vs. without ectopic DDX1 expression (*N* = 3 independent replicates; genes with significantly lower expression, blue; genes with significantly higher expression, red, genes known to be regulated by mTORC1 signaling, orange). **d,** Western blot of the relative protein expression of mTOR ser2448 phosphorylation and P70-S6K Thr389 phosphorylation in NGP cell after inducible expression of DDX1 (1000ng/ml doxycycline treatment for 48 hours). Cells transduced with an empty vector served as negative control. **e+f,** Western blot of mTOR phosphorylation at ser2448 and P70-S6K phosphorylation at Thr389 in IMR5/75 (e) and CHP134 (f) cells expressing shRNAs targeting DDX1 compared to cells expressing a shRNA targeting GFP based on the immunoblot shown in Figure 4f*.*


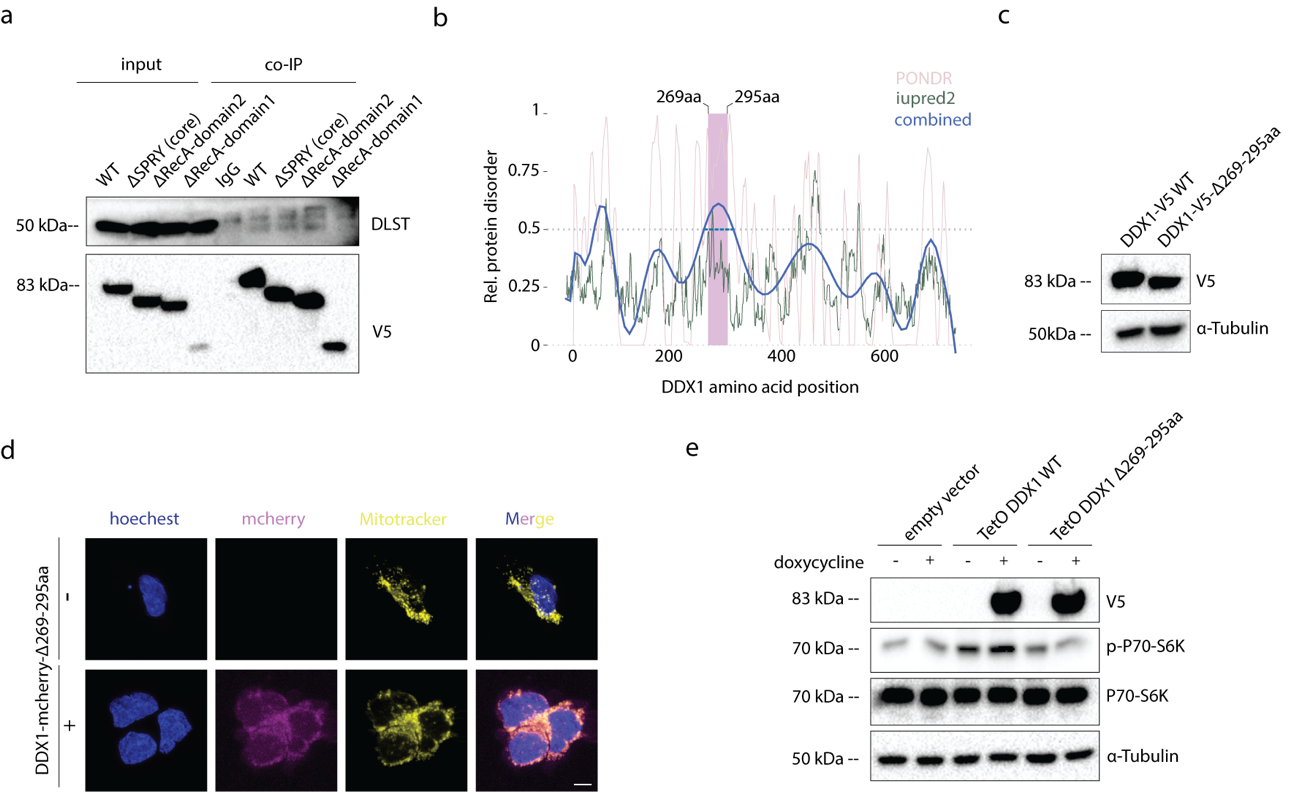


**Supplementary Figure S7. DDX1 interacts with alpha-KGDH complex members and disruption of the DDX1:DLST interaction reduces mTORC1 pathway activation. a,** Western immunoblot of V5, DLST and OGDH before and after immunoprecipitation using antibodies directed against V5, DLST, OGDH or non-specific immunoglobulins (IgG) in Kelly cells expressing DDX1-V5 compared to DDX1-ΔSPRY(core), ΔRecA1 or ΔRecA2 truncation mutants, respectively. **b,** Prediction of disordered regions in DDX1 (top) using Predictor of Natural Disordered regions (PONDR, XL1_XT, pink), Iupred2(green) and polynomial fitted model (blue; the position of amino acids 269-295 is marked in purple). Schematic illustration (bottom) of protein domains in DDX1 as well as engineered DDX1 mutants (DDX1-ΔSPRY (core), Δ69-247aa; ΔRecA1, Δ13-472aa; ΔRecA2, Δ493-681aa). **c,** Western immunoblot of DDX1 in KELLY cell after inducible expression of DDX1-V5 compared to DDX1-V5-D269-295aa (1000ng/ml doxycycline treatment for 48 hours). NGP cell transduced with empty vector serve as controls. **d,** Representative confocal fluorescence imaging photomicrographs of KELLY cells inducibly expressing DDX1-mCherry-Δ269-295aa (magenta), in which mitochondria were stained by MitoTracker DeepRed (yellow) and the nucleus is stained by hoechest (blue; scale bar: 6µm). **e,** Western immunoblot of P70-S6K Thr389 phosphorylation after inducible expression of DDX1 vs. DDX1-V5-Δ269-295aa in KELLY cells (1 µg/ml doxycycline treatment for 48 hours). KELLY cell transduced with empty vector serve as a control.


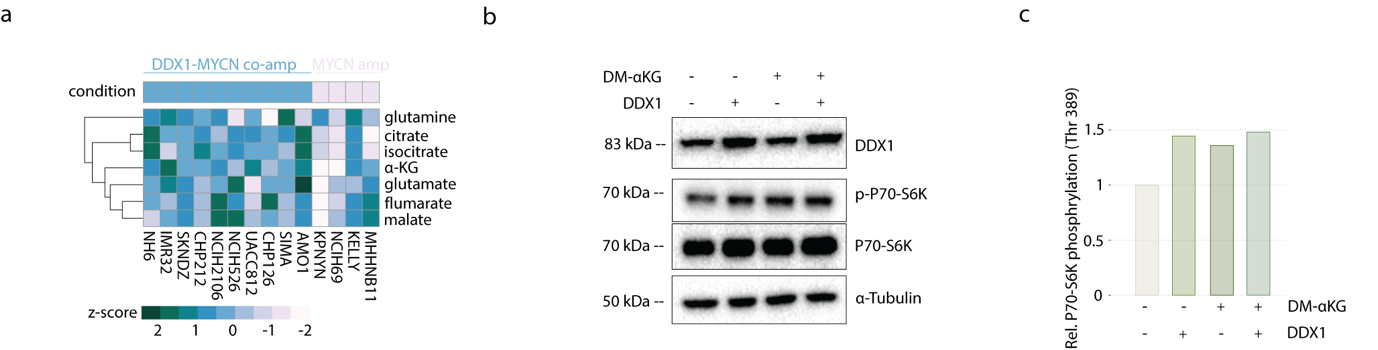


**Supplementary Figure S8. High DDX1 expression is associated with** α**-KG accumulation and OXPHOS reduction. a,** Heatmap of the relative glutamine, citrate, isocitrate, α-KG, glutamate, fumarate and malate concentrations in cancer cell lines with *DDX1*-*MYCN* co-amplification vs. cells with *MYCN* amplifications alone. Cancer cell line metabolism dataset was downloaded from DepMap. **b,** Western immunoblot of P70-S6K Thr389 phosphorylation after incubation of KELLY cells with DM-αKG (2 mM for 48 hours) and inducible DDX1 expression **c,** Quantification of P70-S6K Thr389 phosphorylation after incubation of KELLY cells with DM-αKG (2 mM for 48 hours) and inducible DDX1 expression.


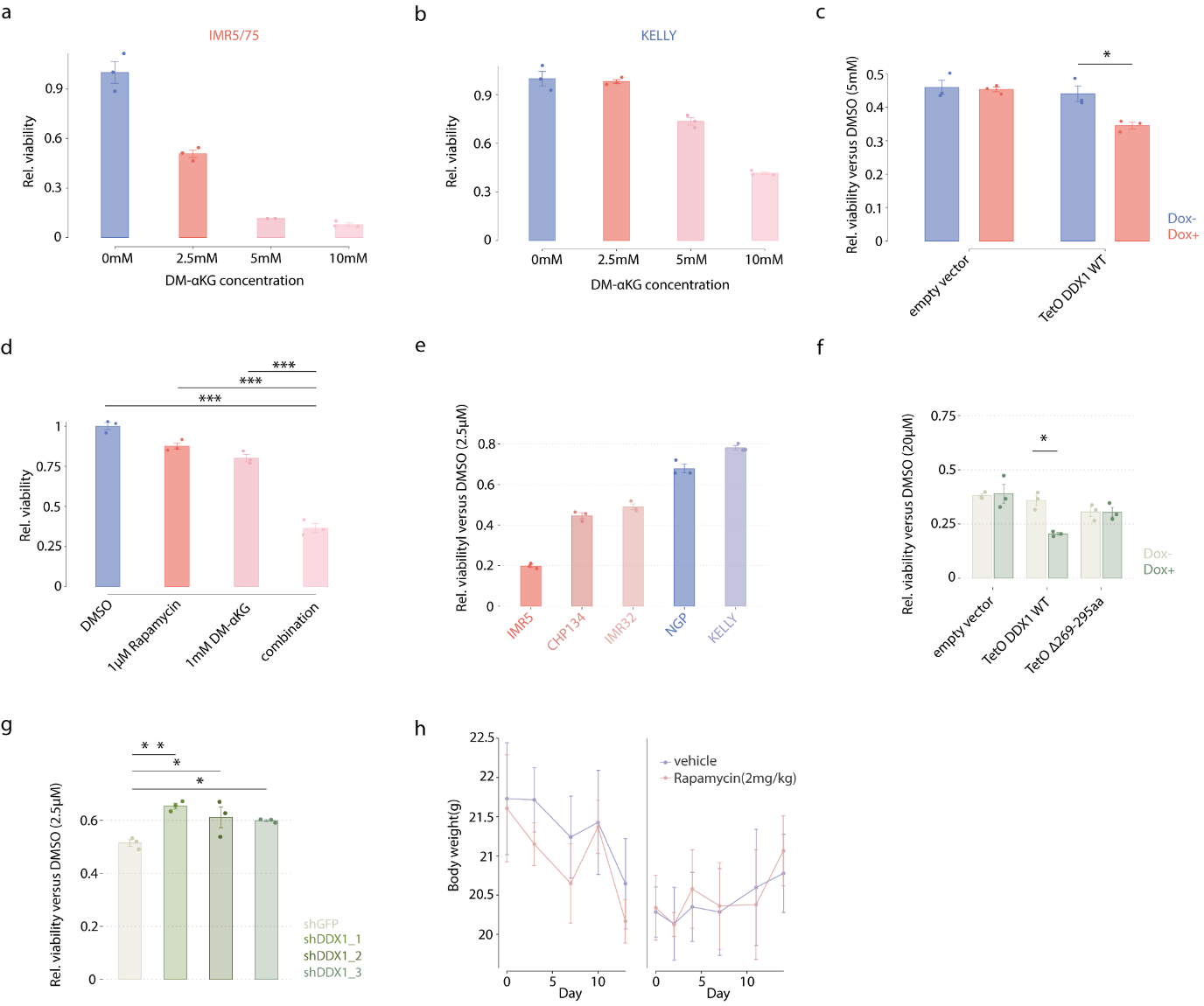


**Supplementary Figure S9. Aberrant DDX1** **expression is associated with increased sensitivity to αKG and pharmacological mTORC1 inhibition. a**, Relative cell viability as measured using MTT assay of IMR5/75 cells harboring a *DDX1-MYCN* co-amplification treated with different concentrations of DM-αKG (0, 2.5, 5 and 10 mM, 72 hours). **b**, Relative cell viability as measured using MTT assay of KELLY cells only harboring a *MYCN* amplification treated with different concentrations of DM-αKG (0, 2.5, 5 and 10 mM, 72 hours)

**c**, Relative cell viability as measured using MTT assay of KELLY cells expressing DDX1 compared to KELLY cells transduced with an empty vector after treatment with DM-αKG (5mM for 72 hours; Welch’s t-test, *P* = 0.03972; Data are shown as mean ± s.e., *N* = 3). **d,** Relative cell viability as measured using MTT assay of IMR5/75 cells after treatment with DM-αKG (1mM) alone, rapamycin (1µM) alone or combination of both compared to DMSO vehicle control treated cells (Welch’s t-test; *P* =2.0e-7, 8.8e-7 and 3.3e-7 for DM-αKG, rapamycin or combination, respectively). Data are shown as mean ± s.e. (*N* = 3). **e**, Relative cell viability as measured using MTT assay of neuroblastoma cell lines with *DDX1*-*MYCN* co-amplification compared to cells with *MYCN* amplification alone after treatment with rapamycin (2.5µM for 72 hours). **f**, Relative cell viability as measured using MTT assay of NGP cells expressing DDX1 compared to NGP cells expressing DDX1 Δ269-295aa after treatment with rapamycin (2.5µM for 72 hours). NGP cells transduced with an empty vector served as negative control. (Welch’s t-test, *P* = 0.01592; Data are shown as mean ± s.e., *N* = 3). **g**, Relative cell viability as measured using MTT assay of CHP134 cells expressing shRNAs targeting DDX1 or GFP (negative control) after treatment with rapamycin (2.5µM for 72 hours). (Pairwise t-test adjusted by Benjamini-Hochberg correction, *P* = 0.01, 0.037 and 0.046 for the three independent shRNAs, respectively; Data are shown as mean ± s.e., *N* = 3). **h**, Mouse body weight during treatment with rapamycin or vehicle.
